# Supplementary material for: Mutant GGGGCC RNA prevents YY1 from binding to Fuzzy promoter which stimulates Wnt/β-catenin pathway in C9ALS/FTD
Source: Nat Commun. 2023 Dec 18;14:8420. doi: 10.1038/s41467-023-44215-w (PMC10728118; doi:10.1038/s41467-023-44215-w)
Supplement: Supplementary file 1 — Supplementary information [file 41467_2023_44215_MOESM1_ESM.pdf]

Supplementary information

Mutant GGGGCC RNA prevents YY1 from binding to *Fuzzy* promoter which stimulates Wnt/β-catenin pathway in C9ALS/FTD

Zhefan Stephen Chen[1,2], Mingxi Ou[1], Stephanie Taylor[2], Ruxandra Dafinca[2,3], Shaohong Isaac Peng[1], Kevin Talbot[2,3,*] & Ho Yin Edwin Chan[1,4,*]

[1]School of Life Sciences, Faculty of Science, The Chinese University of Hong Kong, Shatin, N.T., Hong Kong SAR, China

[2]Oxford Motor Neuron Disease Centre, Nuffield Department of Clinical Neurosciences, John Radcliffe Hospital, University of Oxford, Oxford OX3 9DU, UK

[3]Kavli Institute for Nanoscience Discovery, University of Oxford, Dorothy Crowfoot Hodgkin Building, South Parks Road, Oxford OX1 3QU, UK

[4]Gerald Choa Neuroscience Institute, The Chinese University of Hong Kong, Shatin, N.T., Hong Kong SAR, China

*Correspondence: kevin.talbot@ndcn.ox.ac.uk (K.T.), hyechan@cuhk.edu.hk (H.Y.E.C.)

Supplementary Fig. 1

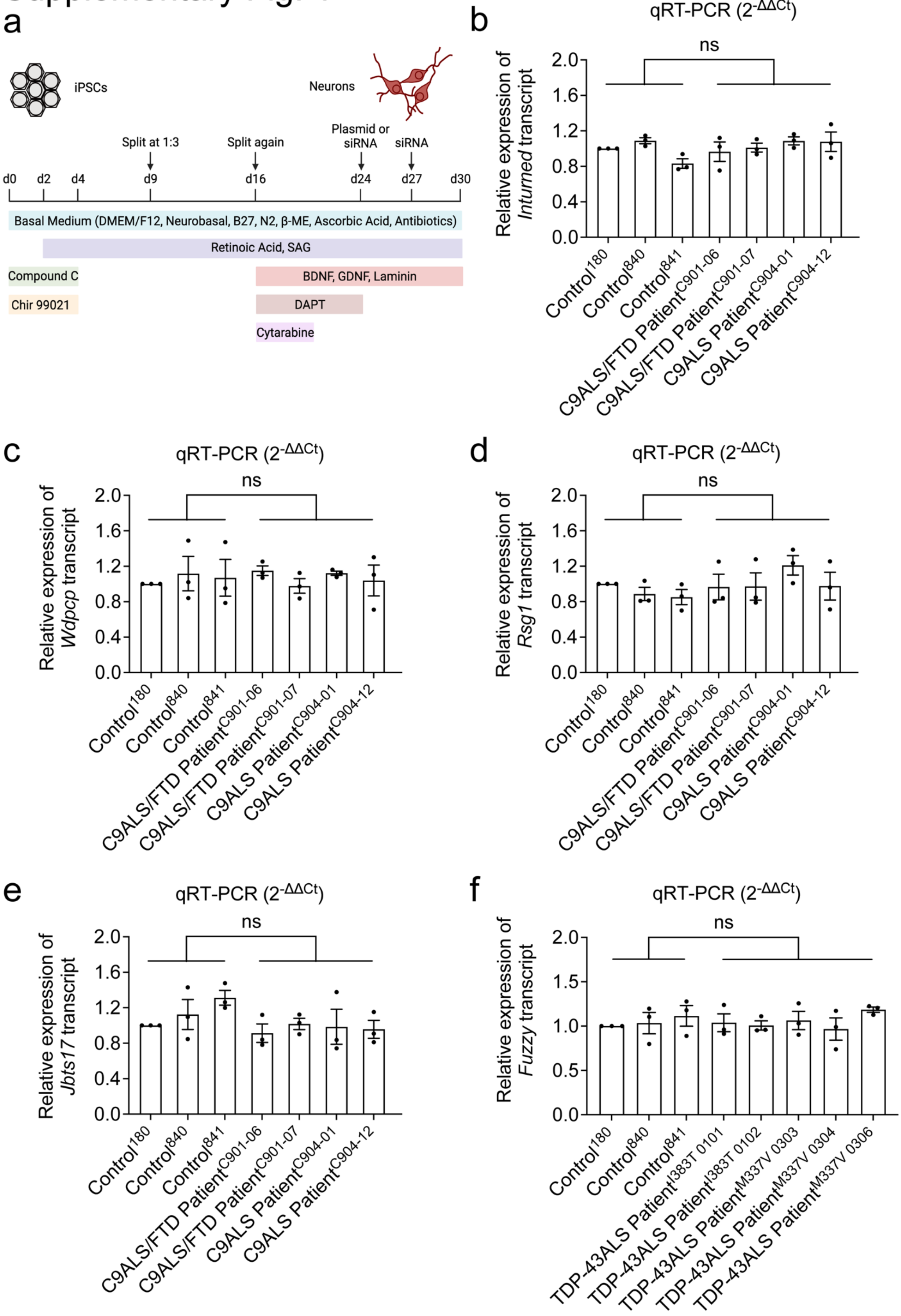

Supplementary Fig. 1 Examination of the expression of CPLANE genes in iPSC-derived spinal motor neurons carrying *C9orf72* or *TARDBP* mutation.

a Schematic representation of the protocol used for the differentiation of iPSCs into spinal motor neurons. **b-e** The expression of *Inturned* (**b**), *Wdpcp* (**c**), *Rsg1* (**d**) and *Jbts17* (**e**) was not altered in disease iPSCs-derived spinal motor neurons compared to the healthy control neurons. **f** The healthy control iPSCs and disease iPSCs with different ALS-causing *TARDBP* mutations were differentiated to spinal motor neurons. The disease neurons' *Fuzzy* transcript level showed no difference from that of healthy control neurons. One-way ANOVA followed by *post hoc* Tukey's test was used in panels 1b-1f. The exact *P* values are listed in Supplementary Table 7. n = 3 biologically independent experiments and data is presented as mean $\pm$ S.E.M. The illustration in panel 1a was created using BioRender.com. Source data are provided as a Source Data file.

Supplementary Fig. 2

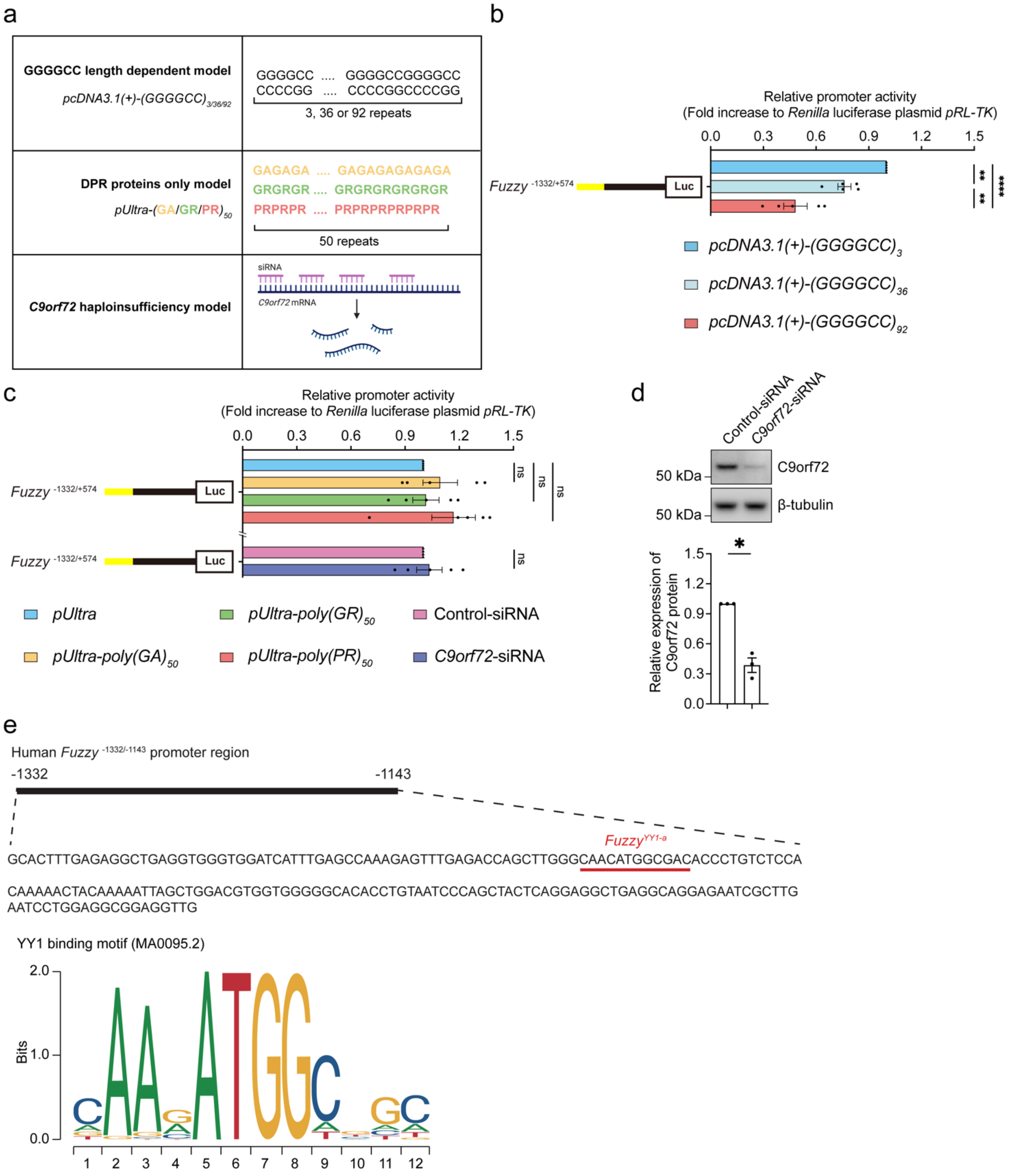

Supplementary Fig. 2 Expression of DPR proteins and reduction of C9orf72 protein level do not affect *Fuzzy* promoter activity.

a Illustration of the different models used in the luciferase assay. The "GGGGCC length dependent model" represents three constructs bearing 3, 36, and 92 pure GGGGCC repeats in length. The "DPR

proteins only model" represents the constructs harbouring codon-optimised nucleotide sequences that produce poly(GA)$_{50}$, poly(GR)$_{50}$, and poly(PR)$_{50}$ proteins, respectively. The sequences are listed in Supplementary Table 2. The "*C9orf72* haploinsufficiency model" indicates using *C9orf72*-targeting siRNA that reduces the C9orf72 protein level. **b** The downregulation of *Fuzzy*$^{-1332/+574}$ promoter activity was GGGGCC repeat length dependent. **c** Downregulation of *Fuzzy*$^{-1332/+574}$ promoter activity was not detected in cells expressing poly(GA)$_{50}$, poly(GR)$_{50}$ or poly(PR)$_{50}$, nor in cells with *C9orf72* knocked down. **d** Reduction of C9orf72 protein level was detected in SK-N-MC cells transfected with *C9orf72*-siRNA. **e** The YY1 (*Fuzzy*$^{YY1\text{-}a}$) transcriptional factor binding site was identified within *Fuzzy*$^{-1332/-1143}$ promoter region. The diagram of YY1 binding motif (MA0095.2) was downloaded from the JASPAR database. One-way ANOVA followed by *post hoc* Tukey's test was used in panel 2b, while one-way ANOVA followed by *post hoc* Dunnett's test was used for the comparisons among different DPR protein constructs in panel 2c. Two-tailed unpaired Student's *t*-test was used in panels 2c and 2d. The exact *P* values are listed in Supplementary Table 7. For panel 2d, n = 3 biologically independent experiments. For panels 2b and 2c, n = 5 biologically independent experiments. Data is presented as mean $\pm$ S.E.M. The illustrations in panels 2a and 2e were created using Adobe Illustrator. Source data are provided as a Source Data file.

Supplementary Fig. 3

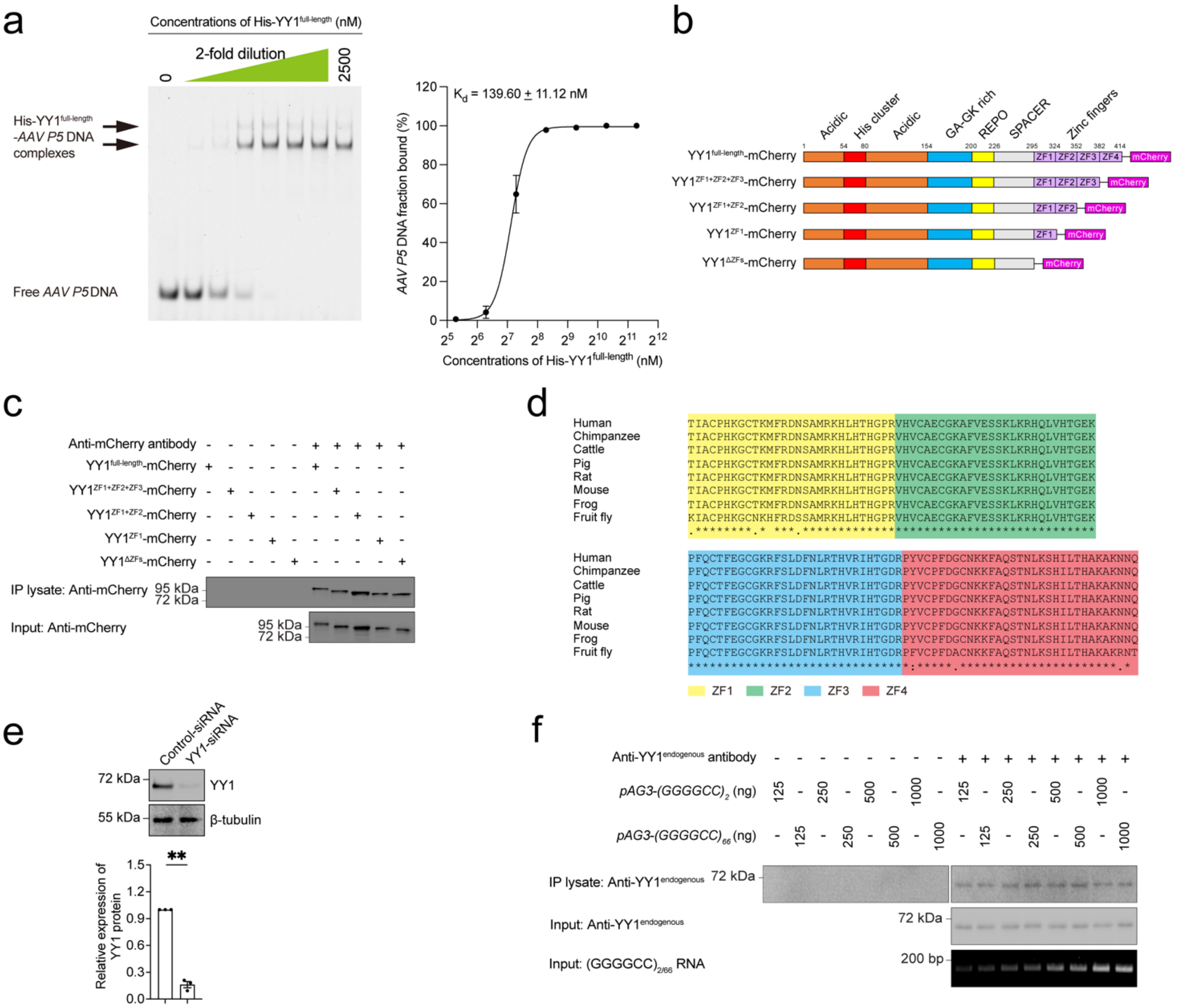

Supplementary Fig. 3 Data related to main Fig. 2.

a YY1 bound to *AAV P5* DNA. The R^2 of the YY1–*AAV P5* DNA binding curve is 0.9872. **b** Illustration of the full-length YY1 protein and a series of mutant YY1 proteins with ZFs sequentially deleted. **c** This panel is supplementary to main Fig. 2b. Anti-mCherry antibody was used for the immunoprecipitation of exogenous full-length and ZF-deleted YY1-mCherry proteins. The presence of these proteins in the "Input" and "IP lysate" samples was detected by immunoblotting. **d** The YY1 ZFs are highly conserved across different species. In particular, the ZF2 and ZF3 are identical in different species. **e** Reduction of YY1 protein level was detected in *YY1*-siRNA-transfected SK-N-MC cells. **f** This panel is supplementary to main Fig. 2f. Anti-YY1 antibody was used for the immunoprecipitation of endogenous YY1 protein. The presence of YY1 protein in the "Input" and "IP

lysate” samples was detected by immunoblotting. The serial overexpression of *(GGGGCC)*$_{2/66}$ RNA in the “Input” sample was detected by RT-PCR. Two-tailed unpaired Student's *t*-test was used in panel 3e. The exact *P* values are listed in Supplementary Table 7. $n = 3$ biologically independent experiments and data is represented as mean ± S.E.M. The sequence of the Cy5-labelled *AAV P5* DNA (20 base pairs in length) probe is listed in Supplementary Table 4. The illustrations in panels 3b and 3d were created using Adobe Illustrator. Source data are provided as a Source Data file.

Supplementary Fig. 4

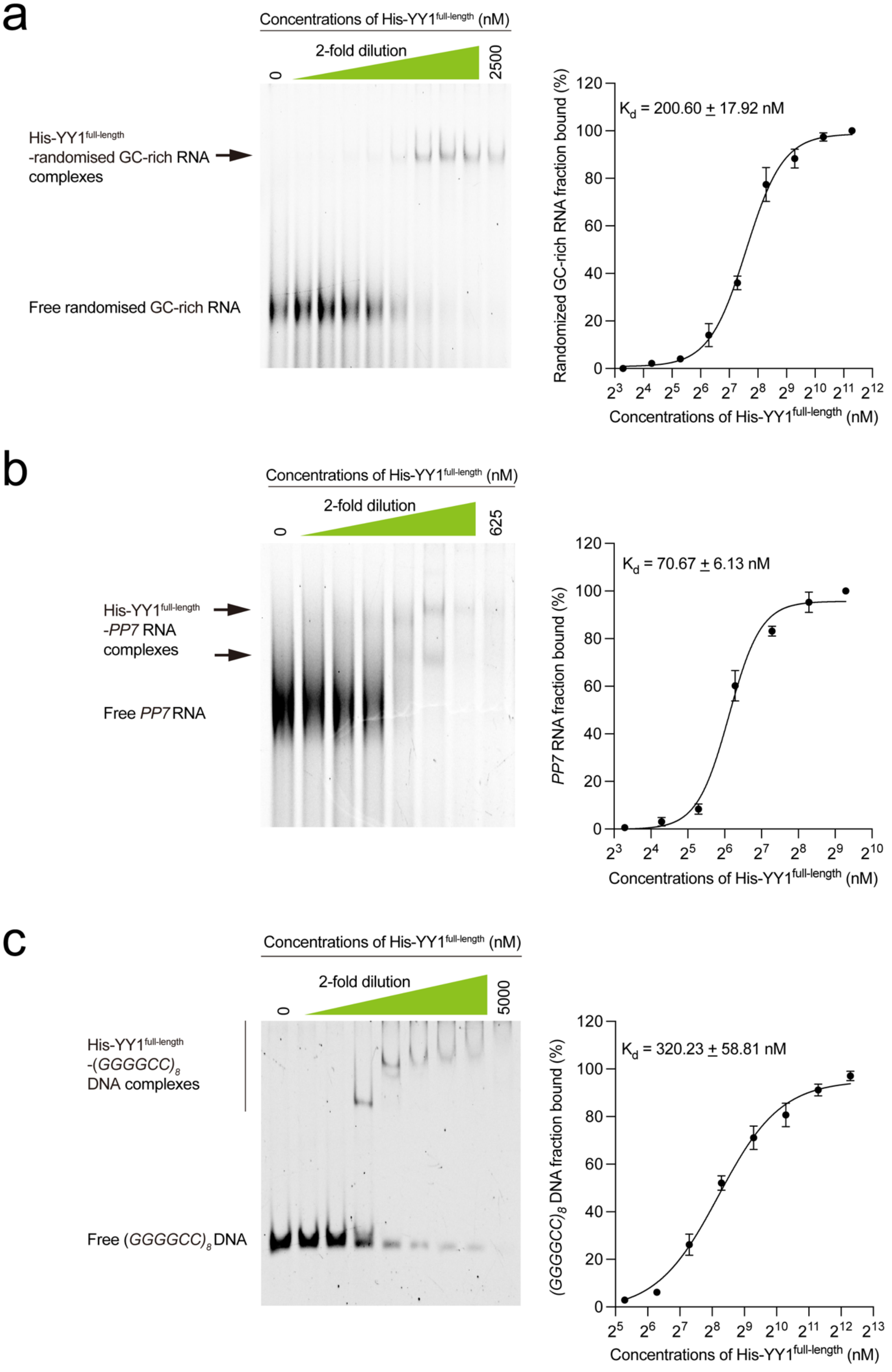

Supplementary Fig. 4 YY1 binds to additional RNA and DNA sequences.

a The YY1 protein bound to randomised GC-rich RNA. The binding affinity is lower than that of YY1–*(GGGGCC)₈* RNA binding (K_d = 46.76 ± 3.42 nM; Fig. 3a). The R^2 of the YY1–randomised GC-rich RNA binding curve is 0.9857. **b** The EMSA was performed to demonstrate the binding between YY1 and *PP7* RNA. The R^2 of the YY1–*PP7* RNA binding curve is 0.9839. **c** Our EMSA result demonstrated the binding between YY1 and *(GGGGCC)₈* DNA. The R^2 of the YY1–*(GGGGCC)₈* DNA binding curve is 0.9805. *n* = 3 biologically independent experiments and data is represented as mean ± S.E.M. The sequences of the Cy5-labelled randomised GC-rich RNA (48 bases in length), *PP7* RNA (100 bases in length) and *(GGGGCC)₈* DNA (48 base pairs in length) probes are listed in Supplementary Table 4. Source data are provided as a Source Data file.

Supplementary Fig. 5

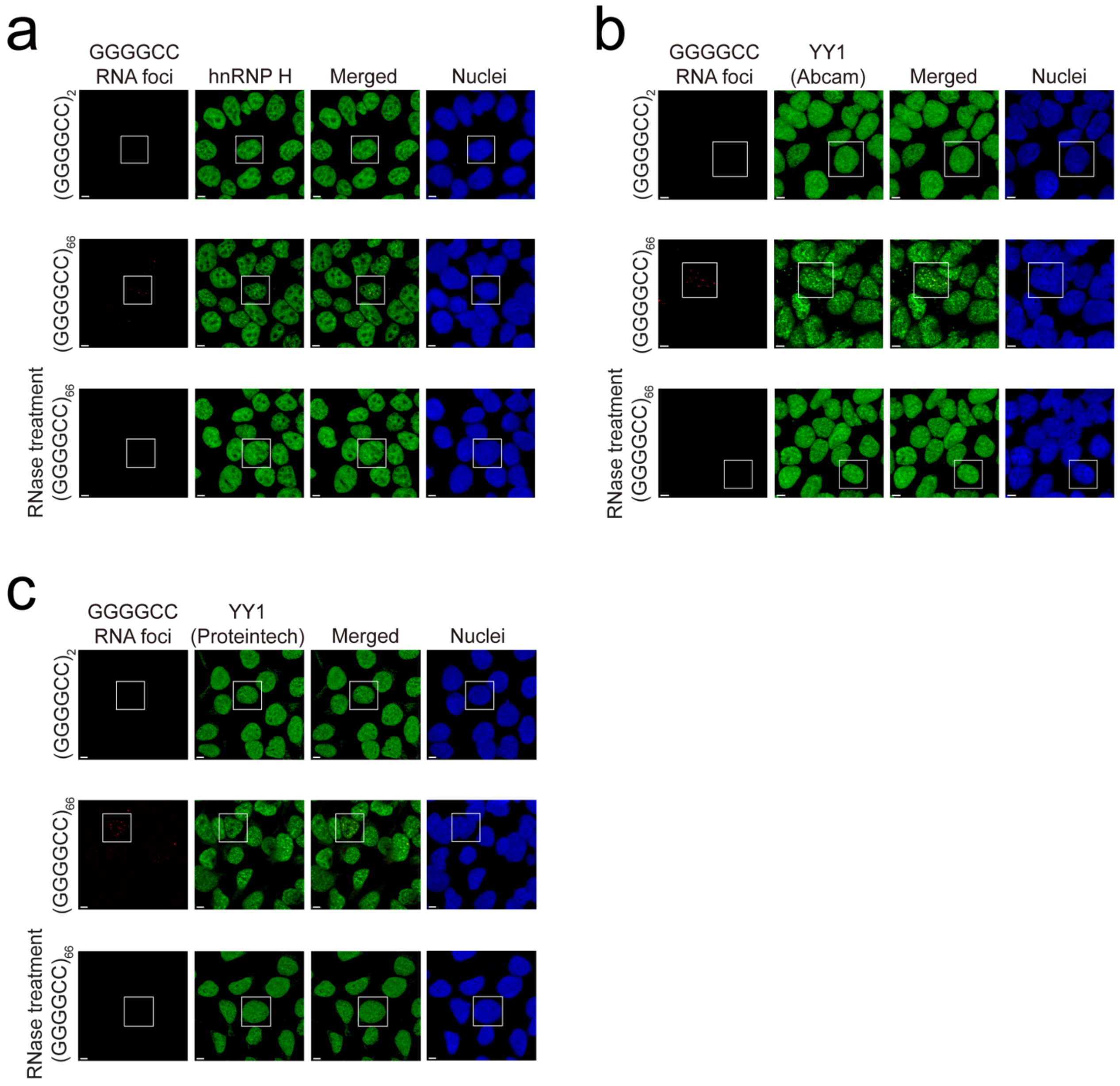

Supplementary Fig. 5 Data related to main Fig. 3.

This figure shows the uncropped images of main Fig. 3c-e. Scale bars: 5 µm.

Supplementary Fig. 6

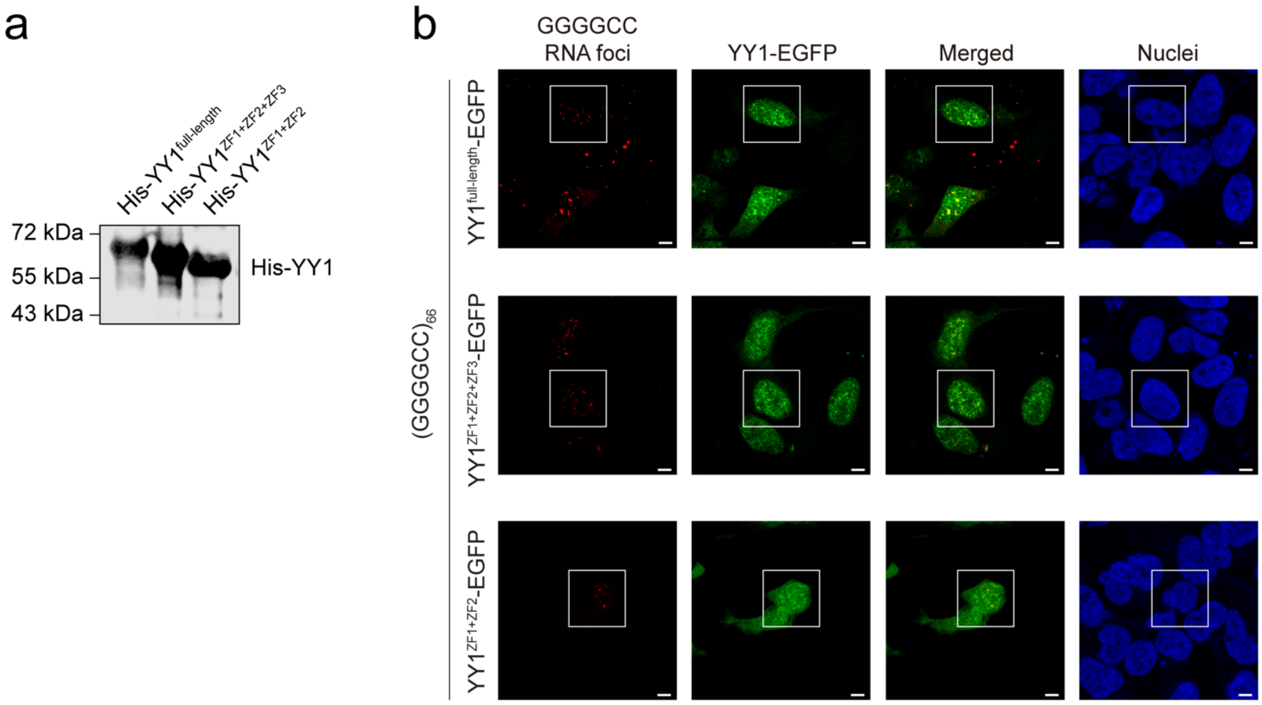

Supplementary Fig. 6 Data related to main Fig. 4.

a Immunoblotting analysis of the recombinant His-tagged YY1$^{\text{full-length}}$, YY1$^{\text{ZF1+ZF2+ZF3}}$ and YY1$^{\text{ZF1+ZF2}}$ proteins used in the EMSA. **b** This figure shows the uncropped images of main Fig. 4b. Scale bars: 5 μm. Source data are provided as a Source Data file.

Supplementary Fig. 7

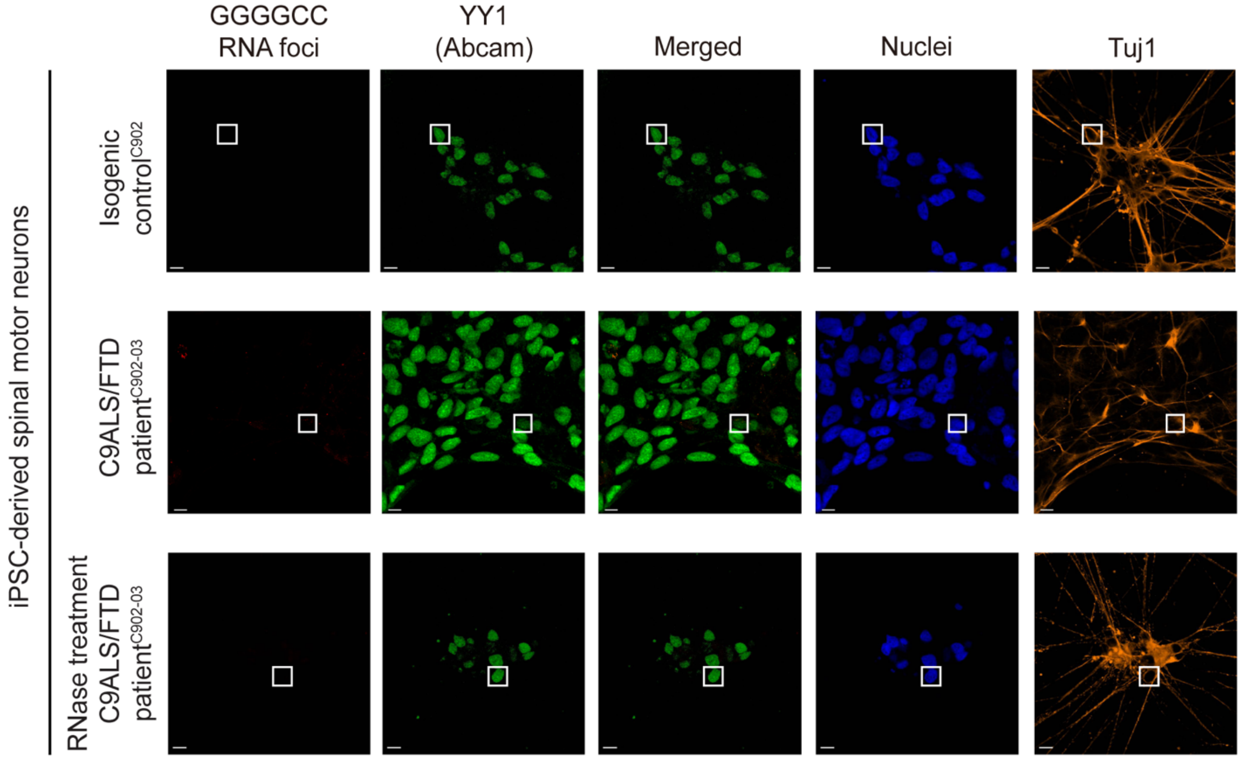

Supplementary Fig. 7 Data related to main Fig. 5a.

This figure shows the uncropped images of main Fig. 5a. Scale bars: 10 µm.

Supplementary Fig. 8

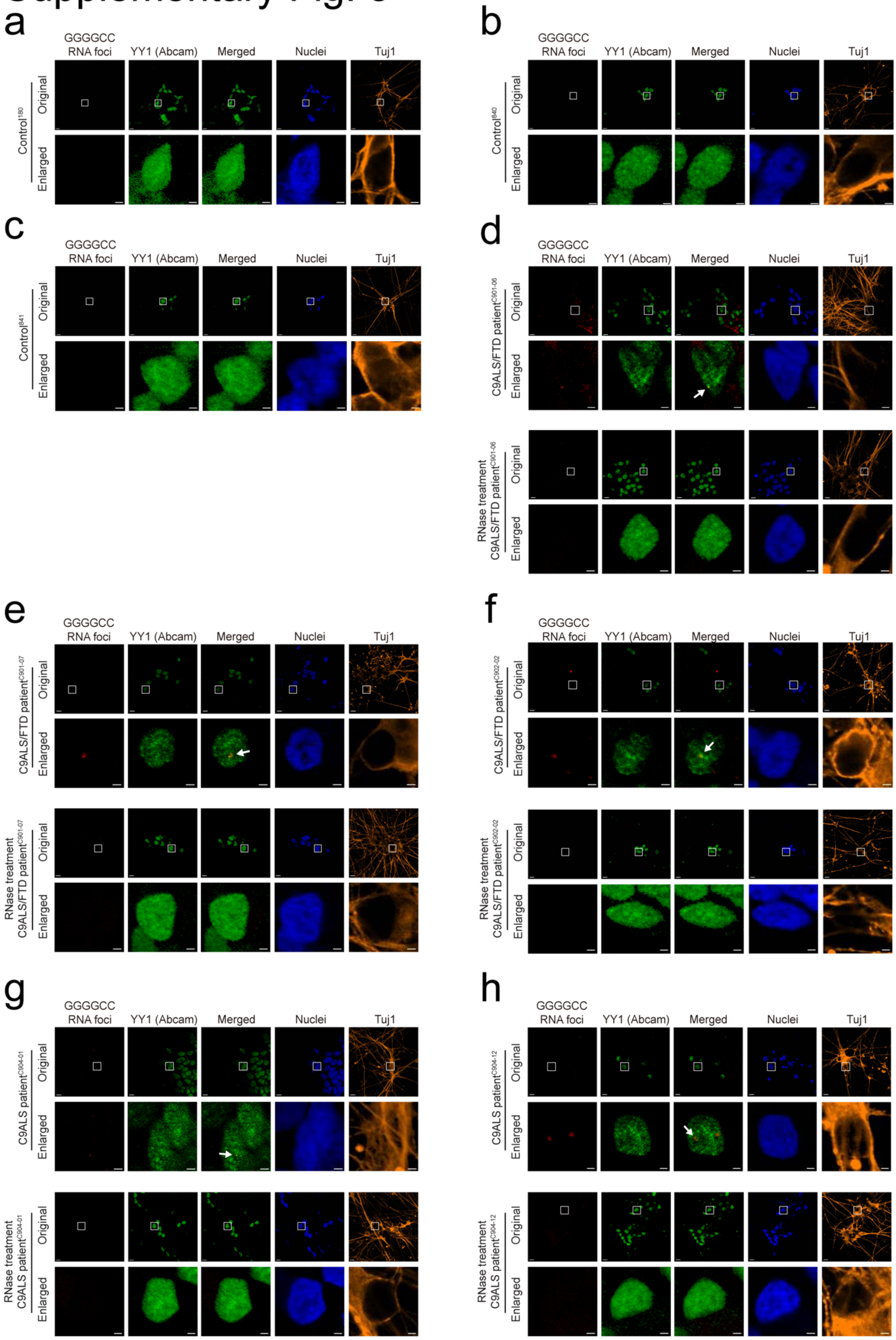

Supplementary Fig. 8 The YY1 protein co-localised with GGGGCC RNA foci in C9ALS/FTD iPSC-derived spinal motor neurons.

a-c No GGGGCC RNA foci was detected in healthy control iPSC-derived spinal motor neurons. **d-h** The recruitment of endogenous YY1 protein (green) to GGGGCC RNA foci (red) was detected in C9ALS/FTD iPSC-derived spinal motor neurons. RNase treatment abolished the GGGGCC RNA foci formation and restored YY1's subcellular localisation. Arrows indicate the co-localisation between the endogenous YY1 protein and GGGGCC RNA foci. The cell nuclei were stained with Hoechst 33342 (blue). Scale bars denote 10 μm and 2 μm in the original and enlarged panels, respectively.

Supplementary Fig. 9

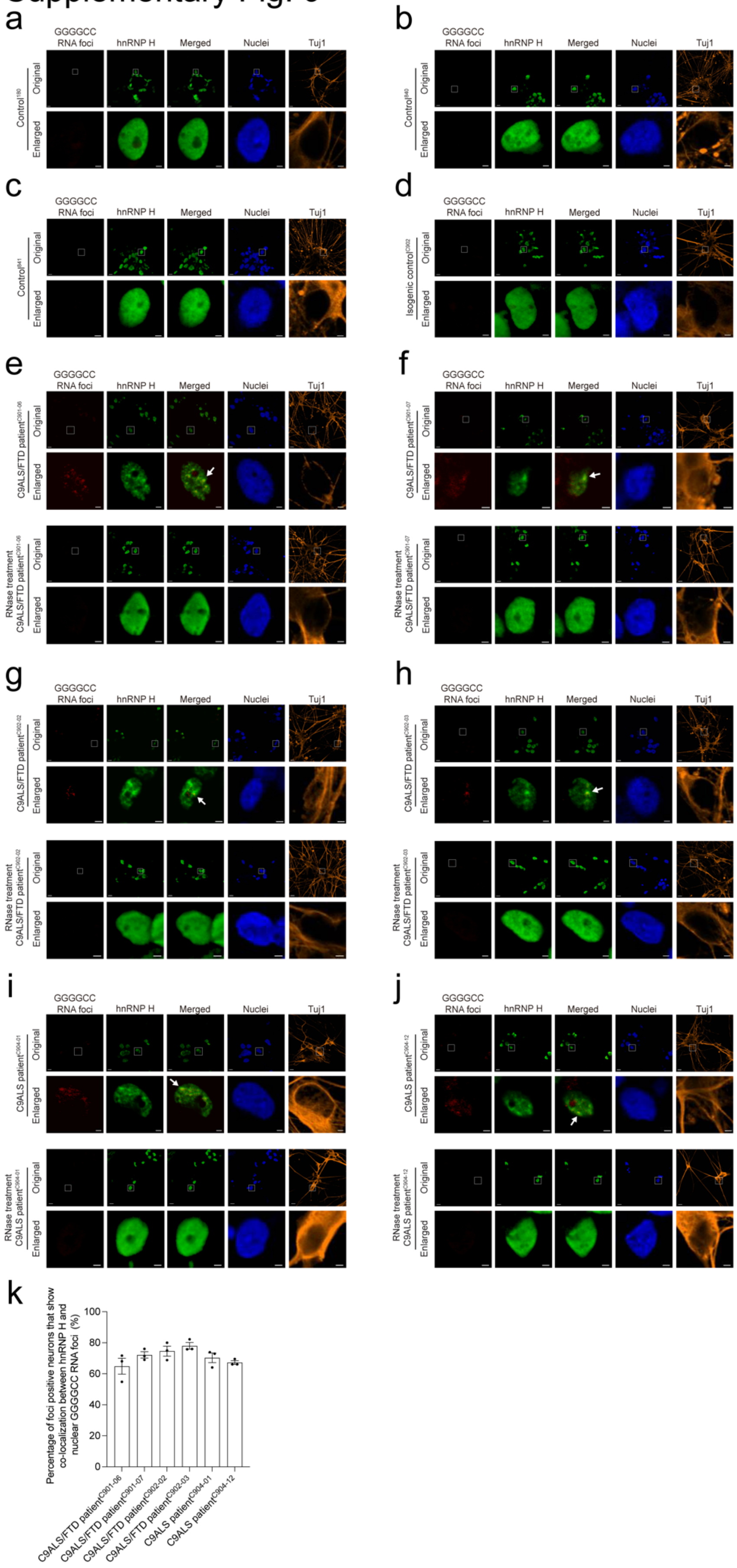

Supplementary Fig. 9 The hnRNP H protein is recruited to GGGGCC RNA foci in C9ALS/FTD iPSC-derived spinal motor neurons.

a-d No GGGGCC RNA foci was detected in the healthy and isogenic control iPSC-derived spinal motor neurons. **e-j** The endogenous hnRNP H protein (green) was recruited to the GGGGCC RNA foci (red) in C9ALS/FTD iPSC-derived spinal motor neurons. Treatment of RNase abolished the formation of GGGGCC RNA foci and restored hnRNP H's subcellular localisation. **k** is the quantification of **a-j**. The number of GGGGCC foci-positive neurons counted over three independent experiments were: C901-06: 187; C901-07: 166; C902-02: 160; C902-03: 174; C904-01: 158; C904-12: 161. Data is presented as mean $\pm$ S.E.M. Arrows indicate the co-localisation between the endogenous hnRNP H protein and GGGGCC RNA foci. The cell nuclei were stained with Hoechst 33342 (blue). Scale bars denote 10 μm and 2 μm in the original and enlarged panels, respectively. Source data are provided as a Source Data file.

Supplementary Fig. 10

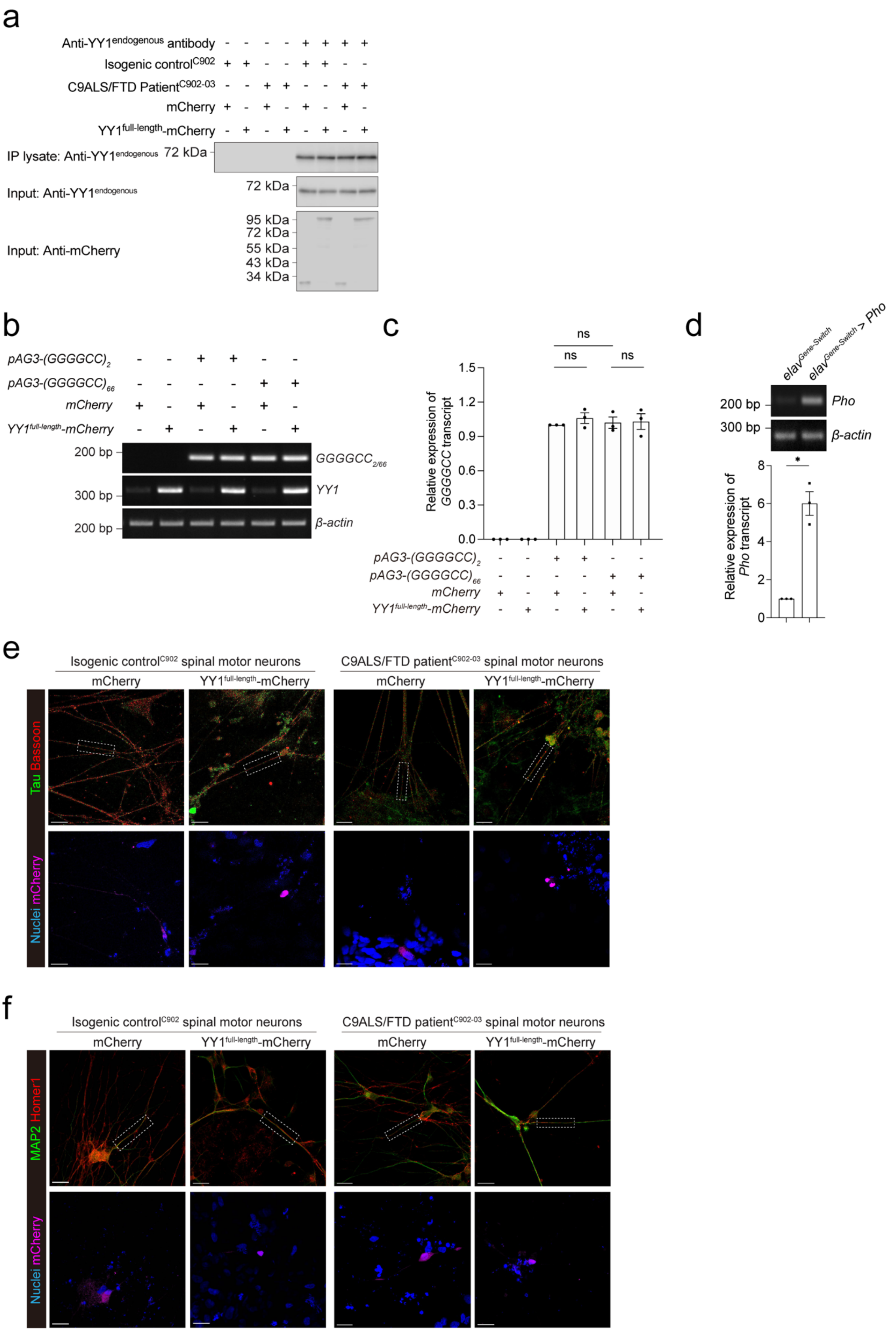

Supplementary Fig. 10 Data related to main Fig. 6.

a This panel is supplementary to main Fig. 6a. Anti-YY1 antibody was used for the immunoprecipitation of endogenous YY1 protein. The presence of YY1 protein in the "Input" and "IP lysate" samples and the presence of mCherry and $YY1^{full\text{-}length}$-mCherry proteins in the "Input" sample were detected by immunoblotting. **b** Overexpression of YY1 did not affect the GGGGCC RNA level in *(GGGGCC)*$_{2/66}$-expressing cells. The GGGGCC RNA was amplified using a pair of GGGGCC primers (Supplementary Table 5) that target the upstream *C9orf72* sequence in the *pAG3-(GGGGCC)*$_{2/66}$ plasmids (Fig. 1c). **c** is the quantification of **b**. **d** Overexpression of *Pho* caused the upregulation of *Pho* level in flies. **e** This panel shows the uncropped images of main Fig. 6f. Scale bars: 20 µm. **f** This panel shows the uncropped images of main Fig. 6h. Scale bars: 20 µm. One-way ANOVA followed by *post hoc* Tukey's test was used in panel 10c. Two-tailed unpaired Student's *t*-test was used in panel 10d. The exact *P* values are listed in Supplementary Table 7. $n = 3$ biologically independent experiments and data is represented as mean ± S.E.M. Source data are provided as a Source Data file.

Supplementary Fig. 11

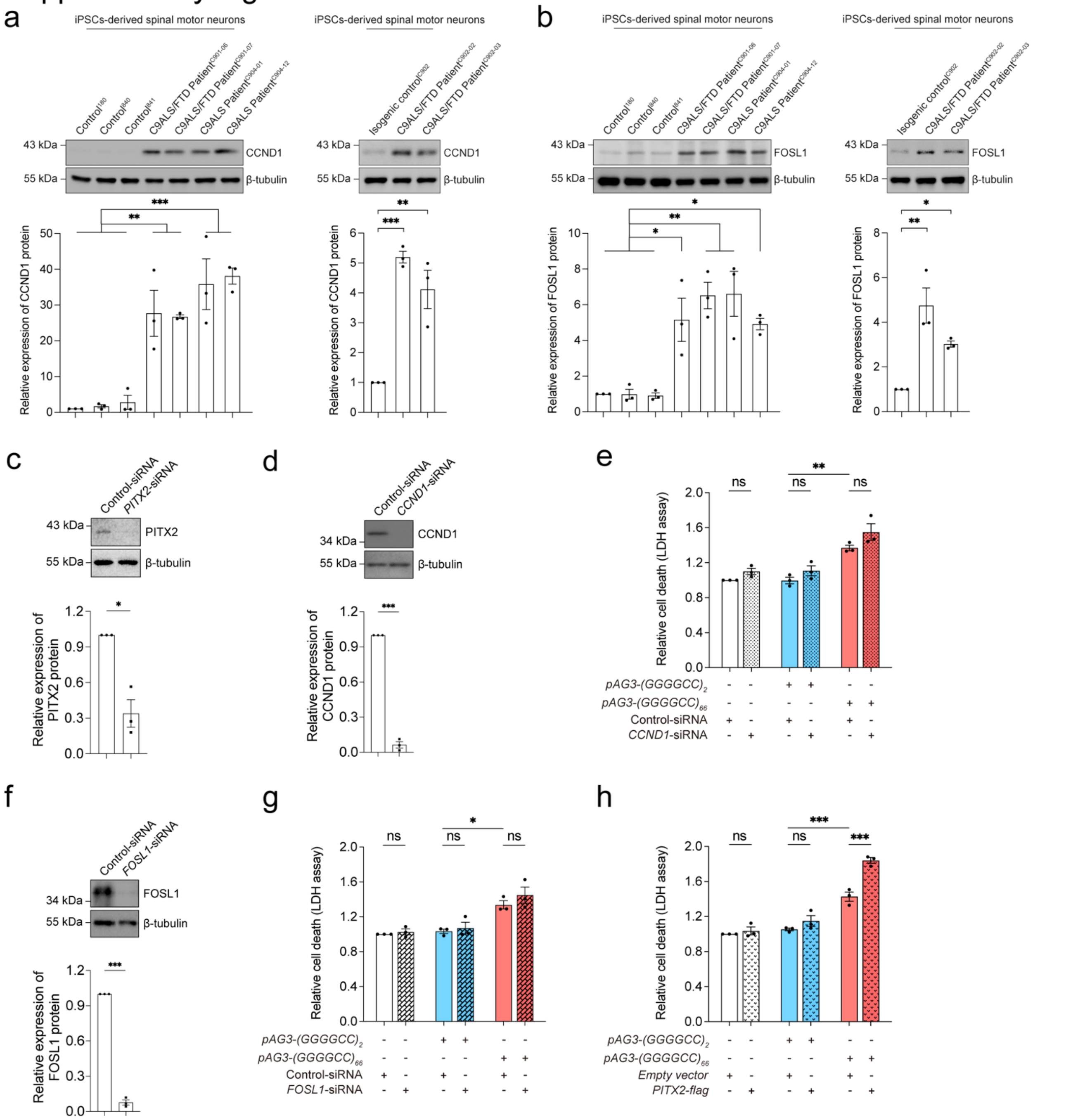

Supplementary Fig. 11 Data related to main Fig. 7.

a, **b** The expression of CCND1 (**a**) and FOSL1 (**b**) was upregulated in C9ALS/FTD iPSC-derived spinal motor neurons compared to the healthy and isogenic control iPSC-derived spinal motor neurons. **c** Reduction of PITX2 protein level was detected in SK-N-MC cells transfected with *PITX2*-siRNA. **d-g** Knockdown of either *CCND1* (**d, e**) or *FOSL1* (**f, g**) did not affect the relative cell death in *(GGGGCC)*$_{66}$-expressing cells. No dominant cytotoxic effect was detected in the untransfected or *(GGGGCC)*$_2$-expressing cells when *CCND1* or *FOSL1* was knocked down. **h** Overexpression of

PITX2 enhanced the *(GGGGCC)*$_{66}$-induced cell death. No dominant cytotoxic effect was detected in the untransfected or *(GGGGCC)*$_{2}$-expressing cells when PITX2 was overexpressed. One-way ANOVA followed by *post hoc* Tukey's test was used in panels 11a, 11b, 11e, 11g and 11h. One-way ANOVA followed by *post hoc* Dunnett's test was used for the comparison between disease and isogenic control neurons in panels 11a and 11b. Two-tailed unpaired Student's *t*-test was used in panels 11c, 11d and 11f. The exact *P* values are listed in Supplementary Table 7. n = 3 biologically independent experiments and data is represented as mean $\pm$ S.E.M. Source data are provided as a Source Data file.

Supplementary Fig. 12

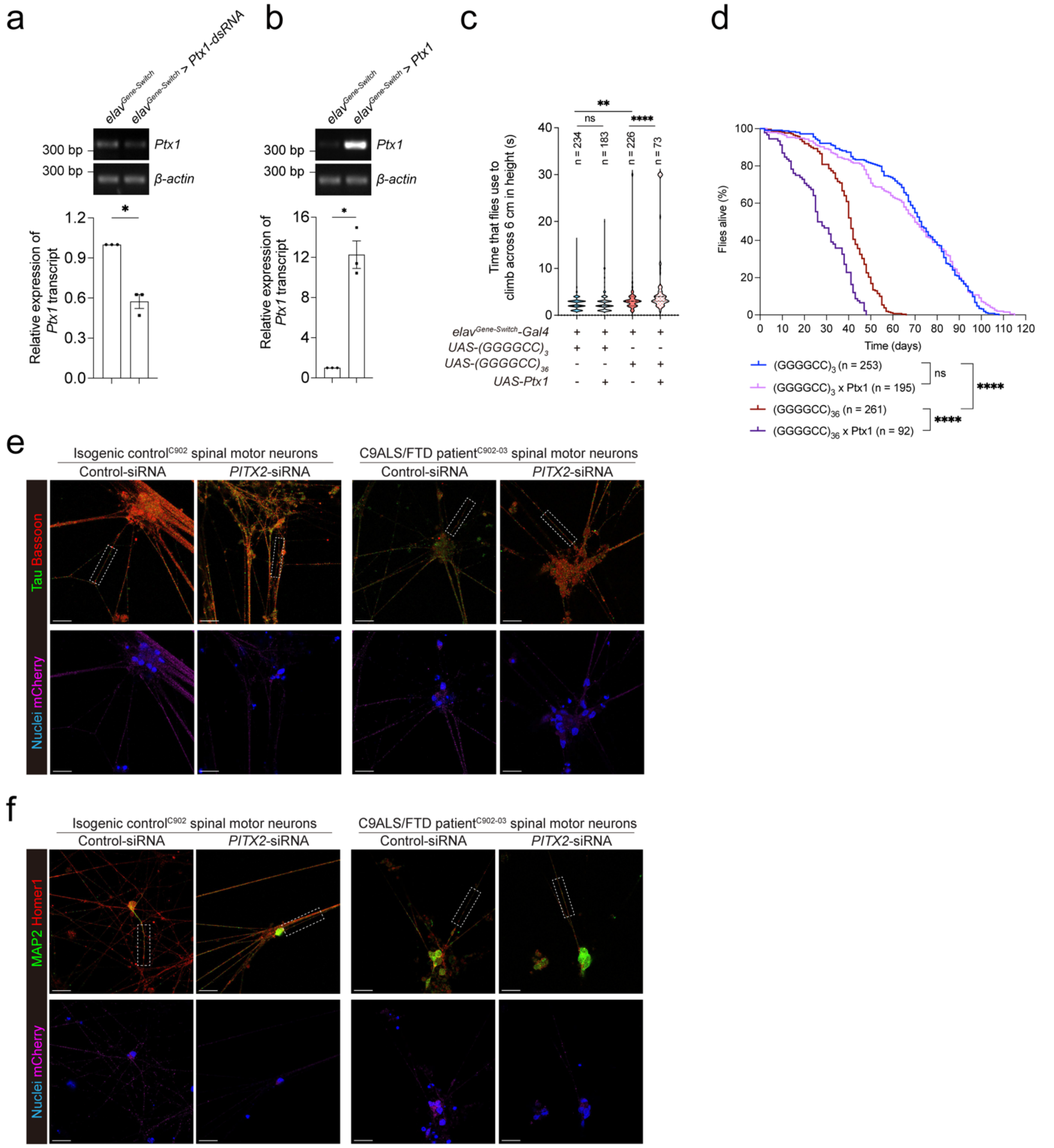

Supplementary Fig. 12 Data related to main Fig. 7 and Supplementary Fig. 11.

a Overexpression of *Ptx1*-dsRNA caused the downregulation of *Ptx1* level in flies. **b-d** Overexpression of *Ptx1* (**b**) enhanced the climbing (**c**) and survival (**d**) defects of $(GGGGCC)_{36}$ flies. The climbing ability and survival probability of $(GGGGCC)_3$ flies were not affected when *Ptx1* was overexpressed. n represents the total number of flies examined over three independent experiments. **e** This panel shows the uncropped images of main Fig. 7h. Scale bars: 20 μm. **f** This panel shows the uncropped images

of main Fig. 7j. Scale bars: 20 μm. Two-tailed unpaired Student's *t*-test was used in panels 12a and 12b. One-way ANOVA followed by *post hoc* Tukey's test was used in panel 12c. Log-rank (Mantel-Cox) test was used in panel 12d. The exact *P* values are listed in Supplementary Table 7. n = 3 biologically independent experiments and data is represented as mean ± S.E.M. Source data are provided as a Source Data file.

Supplementary Fig. 13

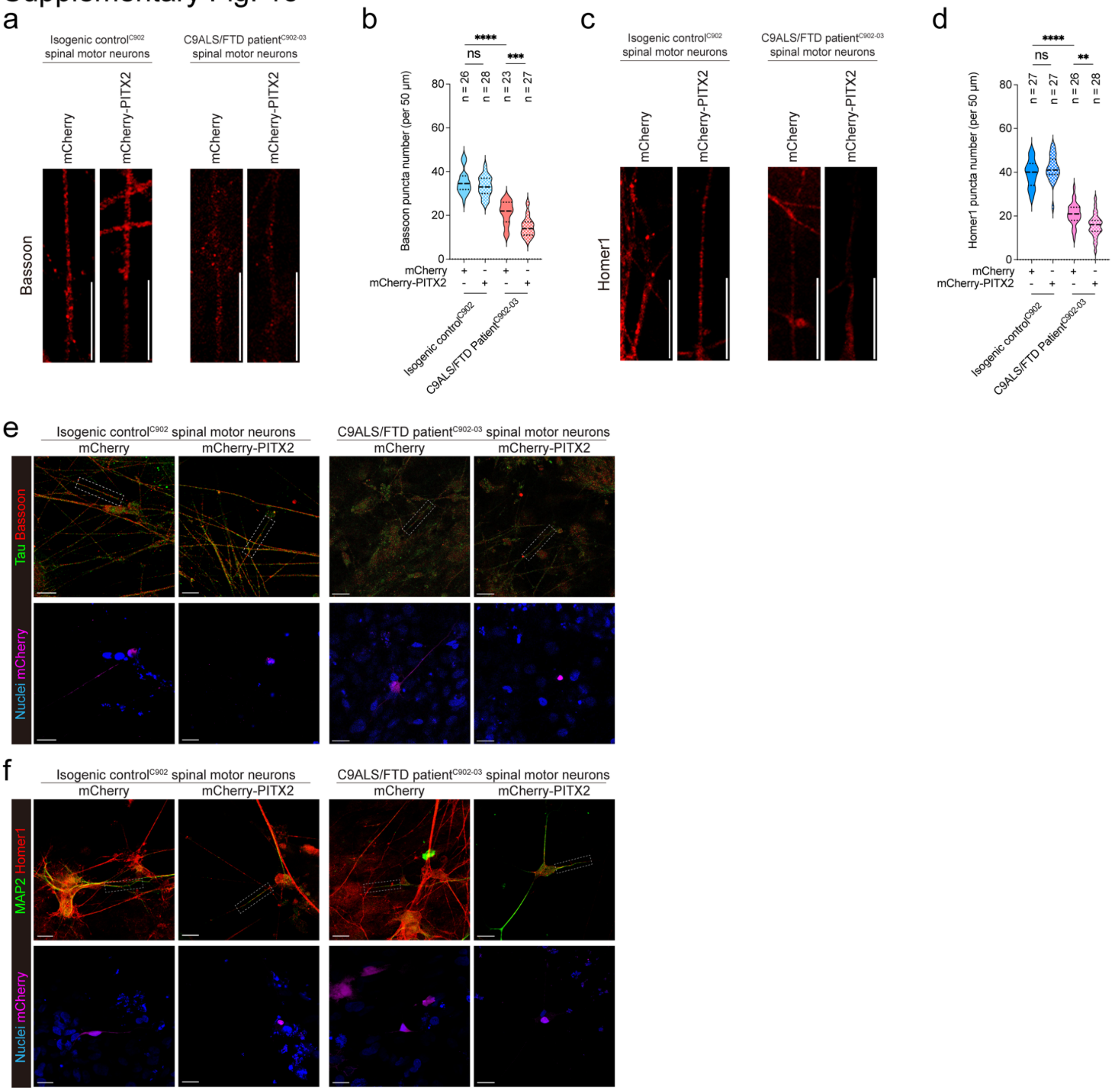

Supplementary Fig. 13 Overexpression of PITX2 enhanced the synaptic defects in C9ALS/FTD spinal motor neurons.

a-d Overexpression of PITX2 enhanced the reduction in Bassoon and Homer1 puncta numbers in C9ALS/FTD iPSC-derived spinal motor neurons. Overexpression of PITX2 did not cause the change in Bassoon and Homer1 puncta numbers in isogenic control iPSC-derived spinal motor neurons. Scale bars: 20 µm. **b** and **d** are the quantifications of **a** and **c**, respectively. *n* represents the total number of neurites examined over three independent experiments. **e** This panel shows the uncropped images of **a**. Scale bars: 20 µm. **f** This panel shows the uncropped images of **c**. Scale bars: 20 µm. One-way

ANOVA followed by *post hoc* Tukey's test was used in panels 13b and 13d. The exact *P* values are listed in Supplementary Table 7. Data is represented as mean $\pm$ S.E.M. Source data are provided as a Source Data file.

Supplementary Fig. 14

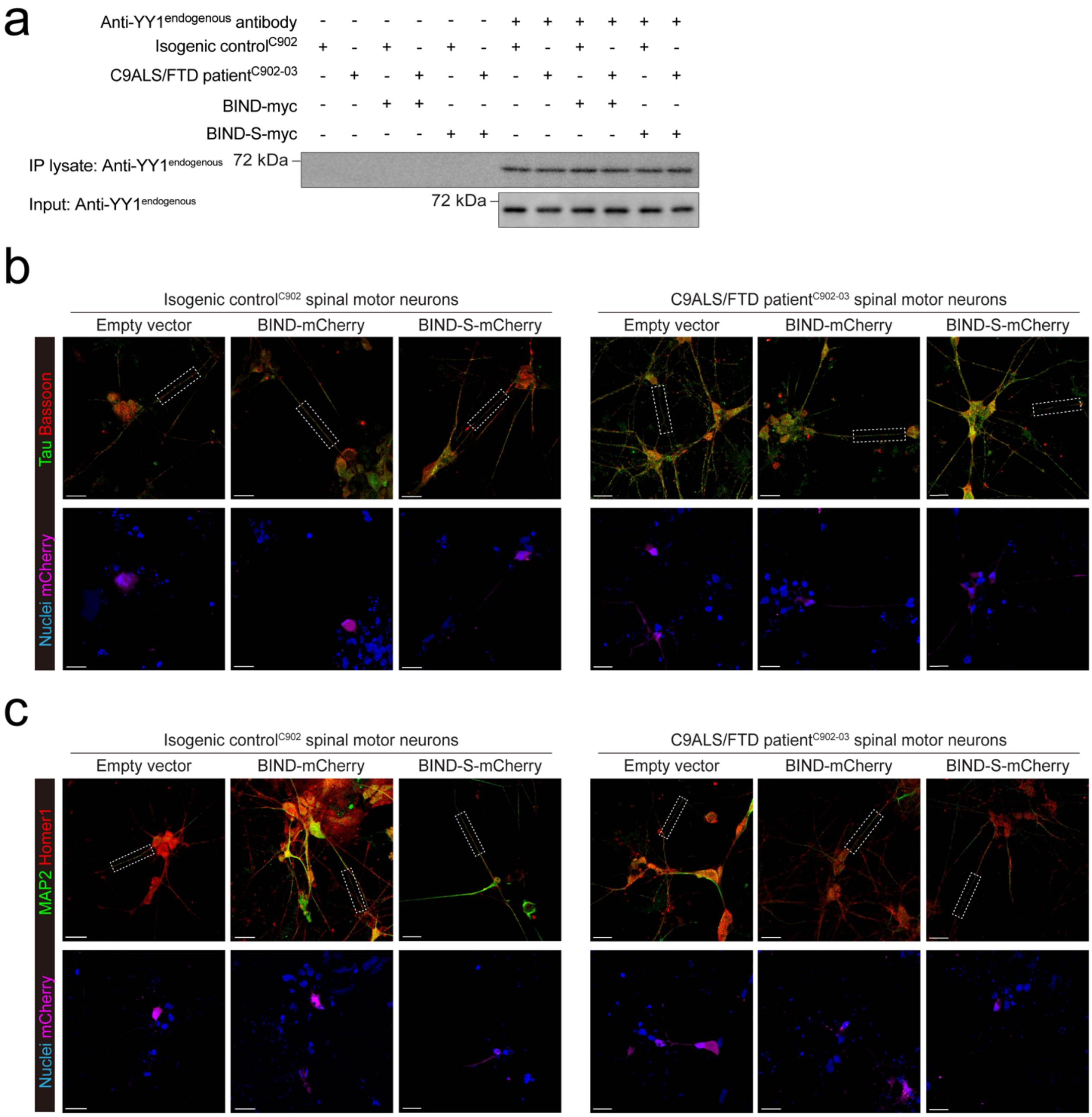

Supplementary Fig. 14 Data related to main Fig. 8.

a This panel is supplementary to main Fig. 8a. Anti-YY1 antibody was used for the immunoprecipitation of endogenous YY1 protein. The presence of YY1 protein in the “Input” and “IP lysate” samples was detected by immunoblotting. **b** This panel shows the uncropped images of main Fig. 8d. Scale bars: 20 µm. **c** This panel shows the uncropped images of main Fig. 8f. Scale bars: 20 µm. Source data are provided as a Source Data file.

Supplementary Fig. 15

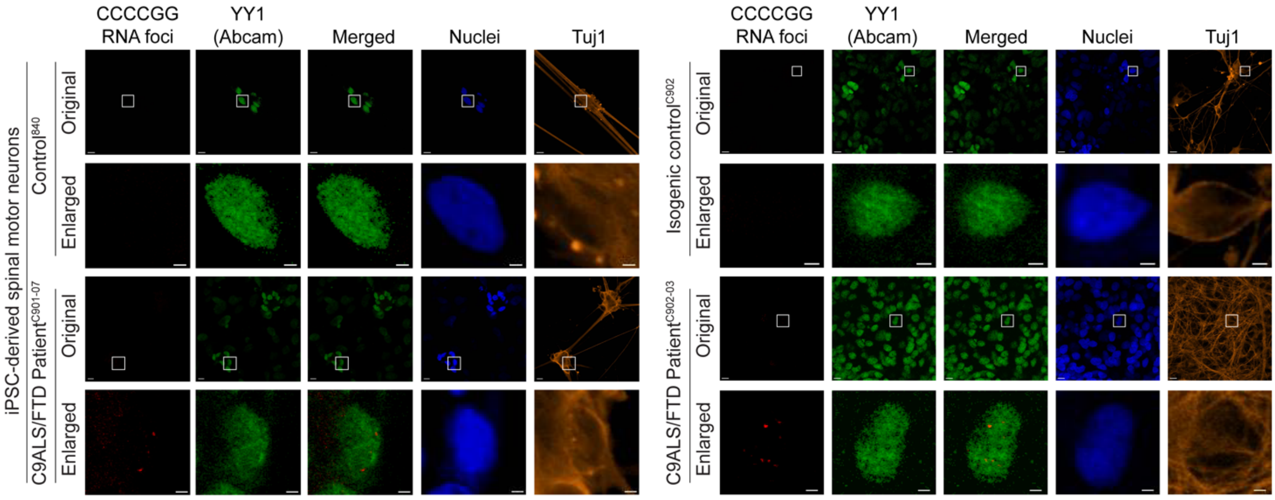

Supplementary Fig. 15 No co-localisation is detected between YY1 and CCCCGG RNA foci in C9ALS/FTD iPSC-derived spinal motor neurons.

The endogenous YY1 protein (green) was not recruited to CCCCGG RNA foci (red) formed in C9ALS/FTD iPSC-derived spinal motor neurons. No CCCCGG RNA foci was detected in the healthy and isogenic control iPSC-derived spinal motor neurons. Scale bars denote 10 μm and 2 μm in the original and enlarged panels, respectively. $n = 3$ biologically independent experiments.

Supplementary Table 1. Demographic information of the control and patient iPSCs used in this study

Line ID	iPSC line	Mutation	Age	Gender	Reference
SFC-180-01-01	Control^{180}	-	60	Female	PMID: 32330447
SFC-840-03-03	Control^{840}	-	67	Female	PMID: 32330447
SFC-841-03-01	Control^{841}	-	36	Male	PMID: 32330447
C901	C9ALS/FTD patient$^{C901\text{-}06}$<br>C9ALS/FTD patient$^{C901\text{-}07}$	*C9orf72*	72	Male	PMID: 32330447
C902	C9ALS/FTD patient$^{C902\text{-}02}$<br>C9ALS/FTD patient$^{C902\text{-}03}$	*C9orf72*	58	Female	PMID: 27097283
C904	C9ALS patient$^{C904\text{-}01}$<br>C9ALS patient$^{C904\text{-}12}$	*C9orf72*	39	Male	PMID: 32330447
C9Edited	Isogenic control^{C902}	-	58	Female	PMID: 32504093
TDP-43-01	ALS patient *TARDBP*$^{I383T\ 0101}$<br>ALS patient *TARDBP*$^{I383T\ 0102}$	*TARDBP*^{I383T}	60	Male	PMID: 32330447
TDP-43-03	ALS patient *TARDBP*$^{M337V\ 0303}$<br>ALS patient *TARDBP*$^{M337V\ 0304}$<br>ALS patient *TARDBP*$^{M337V\ 0306}$	*TARDBP*^{M337V}	57	Male	PMID: 32330447

Supplementary Table 2. The codon-optimised nucleotide sequences used to generate different DPR expression constructs

AgeI-poly(GA)$_{50}$*-BamHI* sequence	ACCGGTATGGGTGCTGGTGCAGGCGCTGGTGCCGGCGCAGGCGCAGGCGCTGGTGCTGGT<br>GCCGGTGCTGGCGCTGGTGCAGGCGCTGGCGCTGGCGCTGGTGCTGGCGCAGGCGCTGGT<br>GCTGGTGCTGGTGCAGGCGCAGGCGCAGGCGCTGGCGCAGGCGCAGGCGCAGGCGCTGGT<br>GCTGGCGCTGGCGCAGGCGCAGGCGCTGGTGCCGGTGCAGGCGCTGGTGCTGGTGCAGGC<br>GCTGGCGCTGGTGCCGGCGCAGGCGCTGGCGCAGGCGCAGGCGCTGGTGCAGGCGCTGGC<br>GCTGGTGCAGAACAAAAACTCATCTCAGAAGAGGATCTGTAAGGATCC
AgeI-poly(GR)$_{50}$*-BamHI* sequence	ACCGGTATGGGGAGAGGTCGTGGGAGAGGCCGCGGTCGAGGGAGAGGCAGGGGACGCGG<br>TAGAGGGAGGGGCCGAGGCAGGGGTAGAGGACGGGGAAGGGGACGTGGCCGGGGGAGA<br>GGAAGAGGAAGAGGGCGAGGGAGAGGTAGAGGTCGTGGACGTGGGAGGGGACGAGGTCG<br>TGGAAGAGGCAGAGGACGTGGTCGAGGTAGAGGCCGAGGAAGAGGTCGGGGGCGTGGGC<br>GTGGAAGGGGTAGGGGTCGTGGTCGCGGACGAGGTAGGGGGCGAGGCCGCGGTAGGGGA<br>AGAGGTAGGGGAAGAGACTACAAAGACGATGACGACAAGTAAGGATCC
AgeI-poly(PR)$_{50}$*-BamHI* sequence	ACCGGTATGCCTCGACCAAGGCCAAGGCCTCGGCCCCGTCCAAGACCCCGTCCGCGTCCTA<br>GGCCAAGGCCACGTCCTCGACCCCGGCCTAGACCCCGACCTCGCCCCAGACCTCGACCTAG<br>ACCACGGCCCCGCCCTAGGCCGCGTCCCCGACCACGTCCAAGACCACGACCACGCCCTCGA<br>CCCAGACCTAGGCCACGACCTAGACCGCGTCCTAGACCTCGACCCAGGCCTCGTCCGAGAC<br>CCAGGCCAAGGCCAAGACCGAGGCCGCGGCCAAGACCACGTCCTAGGCCTAGACCGCGAC<br>CCCGGTACCCATACGATGTTCCAGATTACGCTTAAGGATCC

Supplementary Table 3. The MS2 binding sequence used for the cloning of *pAG3-(GGGGCC)$_{2/66}$-MS2* plasmids

GCGGCCGCGGATCCTAAGGTACCTAATTGCCTAGAAAACATGAGGATCACCCATGTCTGCAGGTCGACTCTAGAAAACATGAGGAT
CACCCATGTCTGCAGTATTCCCGGGTTCATTAGATCCTAAGGTACCTAATTGCCTAGAAAACATGAGGATCACCCATGTCTGCAGGT
CGACTCTAGAAAACATGAGGATCACCCATGTCTGCAGTATTCCCGGGTTCATTAGATCCTAAGGTACCTAATTGCCTAGAAAACATG
AGGATCACCCATGTCTGCAGGTCGACTCTAGAAAACATGAGGATCACCCATGTCTGCAGTATTCCCGGGTTCATTAGATCCTAAGGT
ACCTAATTGCCTAGAAAACATGAGGATCACCCATGTCTGCAGGTCGACTCTAGAAAACATGAGGATCACCCATGTCTGCAGTATTCC
CGGGTTCATTAGATCCTAAGGTACCTAATTGCCTAGAAAACATGAGGATCACCCATGTCTGCAGGTCGACTCTAGAAAACATGAGG
ATCACCCATGTCTGCAGTATTCCCGGGTTCATTAGATCCTAAGGTACCTAATTGCCTAGAAAACATGAGGATCACCCATGTCTGCAG
GTCGACTCTAGAAAACATGAGGATCACCCATGTCTGCAGTATTCCCGGGTTCATTAGATCT

Supplementary Table 4. List of DNA and RNA probes used in this study

**DNA probes**		
**Probe names**	**Manufacturer**	**Sequences**
Cy5-*Fuzzy*$^{YY1\text{-}a}$ DNA	Integrated DNA Technologies	5’-TGGGCAACATGGCGACACCC-3’<br>3’-ACCCGTTGTACCGCTGTGGG-5’
Cy5-*Fuzzy*$^{YY1\text{-}b}$ DNA	Integrated DNA Technologies	5’-AGTCCTAAATGGGGGAGGAG-3’<br>3’-TCAGGATTTACCCCCTCCTC-5’
Cy5-*AAV P5* DNA	Integrated DNA Technologies	5’-AGGGTCTCCATTTTGAAGCG-3’<br>3’-TCCCAGAGGTAAAACTTCGC-5’
Cy5-*(GGGGCC)*$_8$ DNA	Integrated DNA Technologies	5’-GGGGCCGGGGCCGGGGCCGGGGCCGGGGCCGGGGCCGGGGCCGGGGCC-3’<br>3’-CCCCGGCCCCGGCCCCGGCCCCGGCCCCGGCCCCGGCCCCGGCCCCGG-5’
**RNA probes**		
**Probe names**	**Manufacturer**	**Sequences**
Cy5-*(GGGGCC)*$_8$ RNA	Integrated DNA Technologies	5’-GGGGCCGGGGCCGGGGCCGGGGCCGGGGCCGGGGCCGGGGCCGGGGCC-3’
Cy5-randomised GC-rich RNA	Integrated DNA Technologies	5’-GGGCGCGGCGCGCGGGCCGGGGCGGGCGCGCGGGCGGGGCGCGGCGCG-3’
Cy5-*PP7* RNA	Dharmacon	5’-UAAAGUAAGAUAAGGCAAGACAAGGUAAAACGGAACAGAACCGAAGGGAA<br>GAGAAGCAAAGCGAAAGGAAAUAAACAAAAGAAAAAUUCGUAGAAUUCCG-3’
FAM-*(GGGGCC)*$_8$ RNA	Integrated DNA Technologies	5’-CGGGGCCGGGGCCGGGGCCGGGGCCGGGGCCGGGGCCGGGGCCGGGGCCG-3’

Supplementary Table 5. List of primers used in this study

Conventional PCR primers	
**Primer names**	**Sequences (5' to 3')**
GGGGCC-forward	AGTACTCGCTGAGGGTG
GGGGCC-reverse	TAGCGCGCGACTCCTGAG
YY1-forward	TCAGATTCTCATCCCGGTGC
YY1-reverse	CTGTTCTTCAACCACTGTCTC
Pho-forward	TACATTCGAGGGATGCGGAA
Pho-reverse	TGAACAACCACTTTTCCCCG
Ptx1-forward	CACACTTCACCTCGCAACAG
Ptx1-reverse	GTTCTGGTGATGATTGCCGG
β-actin-forward	ATGTGCAAGGCCGGTTTCGC
β-actin-reverse	CGACACGCAGCTCATTGTAG

qPCR primers	
**Primer names**	**Sequences (5' to 3')**
Fuzzy-forward	ACCCCTCAGCCAGTTGTATC
Fuzzy-reverse	AGGATGTCTGTGTGAAGGGG
Inturned-forward	CGCATAGATGAACGGCTAGC
Inturned-reverse	AGCGTTCTTCTGCATGTTGG
Wdpcp-forward	AGCTGGGCCTGATAGACATC
Wdpcp-reverse	CTGATAAAGCACTGGTGGCC
Rsg1-forward	TGCCTCCTTTGAAGACCTCC
Rsg1-reverse	ATGACCATCCTGACGACACC
Jbts17-forward	GACTCCTTCCAACCTGCTCT
Jbts17-reverse	CAGTTTCTGCCCACGTCTTC
YY1-forward	TCAGATTCTCATCCCGGTGC
YY1-reverse	ACTCTTCTTGCCGCTCTTCT
β-actin-forward	CAGCCATGTACGTTGCTATCCAGG
β-actin-reverse	AGGTCCAGACGCAGGATGGCATG

Supplementary Table 6. Summary of the fly genotypes in this study.

**Fig. 6d, 6e**	w; UAS-(GGGGCC)$_{3}$/+; elav$^{Gene\text{-}Switch}$-Gal4/+
	w; UAS-(GGGGCC)$_{3}$/+; elav$^{Gene\text{-}Switch}$-Gal4/Pho
	w; UAS-(GGGGCC)$_{36}$/+; elav$^{Gene\text{-}Switch}$-Gal4/+
	w; UAS-(GGGGCC)$_{36}$/+; elav$^{Gene\text{-}Switch}$-Gal4/Pho
**Fig. 7f, 7g**	w; UAS-(GGGGCC)$_{3}$/+; elav$^{Gene\text{-}Switch}$-Gal4/+
	w; UAS-(GGGGCC)$_{3}$/UAS-Ptx1-dsRNA^{KK108574}; elav$^{Gene\text{-}Switch}$-Gal4/+
	w; UAS-(GGGGCC)$_{36}$/+; elav$^{Gene\text{-}Switch}$-Gal4/+
	w; UAS-(GGGGCC)$_{36}$/UAS-Ptx1-dsRNA^{KK108574}; elav$^{Gene\text{-}Switch}$-Gal4/+
**Supplementary Fig. 12c, 12d**	w; UAS-(GGGGCC)$_{3}$/+; elav$^{Gene\text{-}Switch}$-Gal4/+
	w; UAS-(GGGGCC)$_{3}$/UAS-Ptx1; elav$^{Gene\text{-}Switch}$-Gal4/+
	w; UAS-(GGGGCC)$_{36}$/+; elav$^{Gene\text{-}Switch}$-Gal4/+
	w; UAS-(GGGGCC)$_{36}$/UAS-Ptx1; elav$^{Gene\text{-}Switch}$-Gal4/+

Supplementary Table 7. Summary of the exact *P* values in main and supplementary figures.

Fig. 1a Statistical analysis was performed using one-way ANOVA followed by *post hoc* Tukey's test: 180 vs C901-06, **$P = 0.0016$; 180 vs C901-07, **$P = 0.0032$; 180 vs C904-01, **$P = 0.0011$; 180 vs C904-12, **$P = 0.0065$; 840 vs C901-06, **$P = 0.0023$; 840 vs C901-07, **$P = 0.0048$; 840 vs C904-01, **$P = 0.0017$; 840 vs C904-12, **$P = 0.0099$; 841 vs C901-06, ***$P = 0.0004$; 841 vs C901-07, ***$P = 0.0008$; 841 vs C904-01, ***$P = 0.0003$; 841 vs C904-12, **$P = 0.0017$. Statistical analysis was performed using one-way ANOVA followed by *post hoc* Dunnett's test: C902 vs C902-02, **$P = 0.0090$; C902 vs C902-03, **$P = 0.0083$.

Fig. 1b Statistical analysis was performed using one-way ANOVA followed by *post hoc* Tukey's test: 180 vs C901-06, **$P = 0.0041$; 180 vs C901-07, **$P = 0.0015$; 180 vs C904-01, **$P = 0.0014$; 180 vs C904-12, **$P = 0.0011$; 840 vs C901-06, ****$P < 0.0001$; 840 vs C901-07, ****$P < 0.0001$; 840 vs C904-01, ****$P < 0.0001$; 840 vs C904-12, ****$P < 0.0001$; 841 vs C901-06, **$P = 0.0085$; 841 vs C901-07, **$P = 0.0030$; 841 vs C904-01, **$P = 0.0028$; 841 vs C904-12, **$P = 0.0022$. Statistical analysis was performed using one-way ANOVA followed by *post hoc* Dunnett's test: C902 vs C902-02, **$P = 0.0084$; C902 vs C902-03, **$P = 0.0013$.

Fig. 1d Statistical analysis was performed using two-tailed unpaired Student's *t*-test: $Fuzzy^{-2732/+574}$, ***$P = 0.0008$; $Fuzzy^{-2032/+574}$, *$P = 0.0263$; $Fuzzy^{-1332/+574}$, **$P = 0.0032$; $Fuzzy^{-632/+574}$, $P = 0.1878$.

Fig. 1e Statistical analysis was performed using two-tailed unpaired Student's *t*-test: 3 vs 106-RO, **$P = 0.0032$; 3 + BIND vs 106-RO + BIND, $P = 0.6131$.

Fig. 1f Statistical analysis was performed using two-tailed unpaired Student's *t*-test: $Fuzzy^{-1332/+574}$, ***$P = 0.0003$; $Fuzzy^{-1142/+574}$, $P = 0.6616$; 3 vs 106-RO, $P = 0.5514$.

Fig. 1h Statistical analysis was performed using two-tailed unpaired Student's *t*-test: 2 + wild-type vs 66 + wild-type, **$P = 0.0065$; 2 + YY1-a mutant vs 66 + YY1-a mutant, $P = 0.6013$.

Fig. 1i Statistical analysis was performed using two-tailed unpaired Student's *t*-test: 2 + wild-type vs 66 + wild-type, ***$P = 0.0001$; 2 + YY1-b mutant vs 66 + YY1-b mutant, ***$P = 0.0002$.

Fig. 2b Statistical analysis was performed using one-way ANOVA followed by *post hoc* Tukey's test:

1) $Fuzzy^{YY1\text{-}a}$: $YY1^{full\text{-}length}$ vs $YY1^{ZF1+ZF2}$, ****$P < 0.0001$; $YY1^{full\text{-}length}$ vs $YY1^{ZF1}$, ****$P < 0.0001$; $YY1^{full\text{-}length}$ vs $YY1^{\Delta ZFs}$, ***$P = 0.0002$; $YY1^{ZF1+ZF2+ZF3}$ vs $YY1^{ZF1+ZF2}$, ****$P < 0.0001$; $YY1^{ZF1+ZF2+ZF3}$ vs $YY1^{ZF1}$, ****$P < 0.0001$; $YY1^{ZF1+ZF2+ZF3}$ vs $YY1^{\Delta ZFs}$, ****$P < 0.0001$;

2) $Fuzzy^{YY1\text{-}b}$: $YY1^{full\text{-}length}$ vs $YY1^{ZF1+ZF2+ZF3}$, ****$P < 0.0001$; $YY1^{full\text{-}length}$ vs $YY1^{ZF1+ZF2}$, ***$P = 0.0002$; $YY1^{full\text{-}length}$ vs $YY1^{ZF1}$, ***$P = 0.0002$; $YY1^{full\text{-}length}$ vs $YY1^{\Delta ZFs}$, ***$P = 0.0005$.

Fig. 2c Statistical analysis was performed using one-way ANOVA followed by *post hoc* Dunnett's test: wild-type vs YY1-a mutant, ***$P = 0.0004$; wild-type vs YY1-b mutant, ****$P < 0.0001$.

Fig. 2d Statistical analysis was performed using two-tailed unpaired Student's *t*-test: control-siRNA vs YY1-siRNA, ***$P = 0.0004$.

Fig. 2f Statistical analysis was performed using two-tailed unpaired Student's *t*-test:
1) *Fuzzy*$^{YY1\text{-}a}$: 2 125ng vs 66 125ng, *P* = 0.1727; 2 250ng vs 66 250ng, **P* = 0.0273; 2 500ng vs 66 500ng, **P* = 0.0101; 2 1000ng vs 66 1000ng, ****P* = 0.0006;
2) *Fuzzy*$^{YY1\text{-}b}$: 2 125ng vs 66 125ng, **P* = 0.0335; 2 250ng vs 66 250ng, **P* = 0.0280; 2 500ng vs 66 500ng, **P* = 0.0120; 2 1000ng vs 66 1000ng, ****P* = 0.0001.

Fig. 2g Statistical analysis was performed using two-tailed unpaired Student's *t*-test: 2 125ng vs 66 125ng, *P* = 0.4902; 2 250ng vs 66 250ng, *P* = 0.1052; 2 500ng vs 66 500ng, ***P* = 0.0019; 2 1000ng vs 66 1000ng, *****P* < 0.0001.

Fig. 3g, 3h Statistical analysis was performed using two-tailed unpaired Mann-Whitney *U* test: YY1: *****P* < 0.0001; hnRNP H: *****P* < 0.0001.

Fig. 4c Statistical analysis was performed using one-way ANOVA followed by *post hoc* Tukey's test: YY1$^{full\text{-}length}$-EGFP vs YY1$^{ZF1+ZF2+ZF3}$-EGFP, *P* = 0.7802; YY1$^{full\text{-}length}$-EGFP vs YY1$^{ZF1+ZF2}$-EGFP, ***P* = 0.0064; YY1$^{ZF1+ZF2+ZF3}$-EGFP vs YY1$^{ZF1+ZF2}$-EGFP, **P* = 0.0131.

Fig. 6a Statistical analysis was performed using one-way ANOVA followed by *post hoc* Tukey's test:
1) Fuzzy$^{YY1\text{-}a}$: C902 vs C902 + YY1, **P* = 0.0137; C902 vs C902-03, *****P* < 0.0001; C902-03 vs C902-03 + YY1, ****P* = 0.0003;
2) Fuzzy$^{YY1\text{-}b}$: C902 vs C902 + YY1, ***P* = 0.0046; C902 vs C902-03, *****P* < 0.0001; C902-03 vs C902-03 + YY1, ****P* = 0.0003.

Fig. 6b Statistical analysis was performed using one-way ANOVA followed by *post hoc* Tukey's test:
1) Fuzzy: C902 vs C902 + YY1, *P* = 0.7076; C902 vs C902-03, ***P* = 0.0011; C902-03 vs C902-03 + YY1, ***P* = 0.0027;
2) YY1: C902 vs C902 + YY1, *****P* < 0.0001; C902-03 vs C902-03 + YY1, ****P* = 0.0001.

Fig. 6c Statistical analysis was performed using one-way ANOVA followed by *post hoc* Tukey's test: Empty vector vs Empty vector + YY1, *P* = 0.8939; 2 vs 2 + YY1, *P* = 0.2578; 2 vs 66, ****P* = 0.0004; 66 vs 66 + YY1, ****P* = 0.0007.

Fig. 6d Statistical analysis was performed using one-way ANOVA followed by *post hoc* Tukey's test: 3 vs 3 + Pho, *P* = 0.9863; 3 vs 36, *****P* < 0.0001; 36 vs 36 + Pho, ****P* = 0.0009.

Fig. 6e Statistical analysis was performed using Log-rank (Mantel-Cox) test: 3 vs 3 + Pho, *P* = 0.8489; 3 vs 36, *****P* < 0.0001; 36 vs 36 + Pho, ****P* = 0.0002.

Fig. 6g, 6i Statistical analysis was performed using one-way ANOVA followed by *post hoc* Tukey's test:
1) Bassoon: C902 vs C902 + YY1, *P* = 0.9829; C902 vs C902-03, *****P* < 0.0001; C902-03 vs C902-03 + YY1, *****P* < 0.0001.
2) Homer1: C902 vs C902 + YY1, *P* = 0.4150; C902 vs C902-03, *****P* < 0.0001; C902-03 vs C902-03 + YY1, *****P* < 0.0001.

Fig. 7a Statistical analysis was performed using two-tailed unpaired Student's *t*-test: TOPflash, **P* = 0.0340; FOPflash, *P* = 0.5218.

Fig. 7b Statistical analysis was performed using two-tailed unpaired Student's *t*-test: AXIN2, *P* = 0.9467; BTRC, *P* = 0.5789; CCND1, **P* = 0.0287; CCND2, *P* = 0.2345; DAB2, *P* = 0.1995; FOSL1, **P* = 0.0429; JUN, *P* = 0.7287; MYC, *P* = 0.0792; PITX2, **P* = 0.0295; PPARD, *P* = 0.2785.

Fig. 7c Statistical analysis was performed using two-tailed unpaired Student's *t*-test: AXIN2, *P* = 0.4405; BTRC, *P* = 0.7089; CCND1, **P* = 0.0258; CCND2, *P* = 0.8061; DAB2, *P* = 0.5407; FOSL1, ***P* = 0.0066; JUN, *P* = 0.2427; MYC, *P* = 0.4106; PITX2, **P* = 0.0102; PPARD, *P* = 0.7873.

Fig. 7d Statistical analysis was performed using one-way ANOVA followed by *post hoc* Tukey's test: 180 vs C901-06, *P* = 0.3464; 180 vs C901-07, ***P* = 0.0016; 180 vs C904-01, **P* = 0.0262; 180 vs C904-12, **P* = 0.0272; 840 vs C901-06, *P* = 0.4324; 840 vs C901-07, ***P* = 0.0021; 840 vs C904-01, **P* = 0.0358; 840 vs C904-12, **P* = 0.0372; 841 vs C901-06, *P* = 0.3061; 841 vs C901-07, ***P* = 0.0013; 841 vs C904-01, **P* = 0.0223; 841 vs C904-12, **P* = 0.0231. Statistical analysis was performed using one-way ANOVA followed by *post hoc* Dunnett's test: C902 vs C902-02, **P* = 0.0221; C902 vs C902-03, **P* = 0.0396.

Fig. 7e Statistical analysis was performed using one-way ANOVA followed by *post hoc* Tukey's test: Empty vector vs Empty vector + PITX2-siRNA, *P* = 0.4961; 2 vs 2 + PITX2-siRNA, *P* = 0.1735; 2 vs 66, ****P* = 0.0002; 66 vs 66 + PITX2-siRNA, ***P* = 0.0013.

Fig. 7f Statistical analysis was performed using one-way ANOVA followed by *post hoc* Tukey's test: 3 vs 3 + Ptx1-dsRNA, *P* = 0.8605; 3 vs 36, *****P* < 0.0001; 36 vs 36 + Ptx1-dsRNA, *****P* < 0.0001.

Fig. 7g Statistical analysis was performed using Log-rank (Mantel-Cox) test: 3 vs 3 + Ptx1-dsRNA, *P* = 0.7825; 3 vs 36, *****P* < 0.0001; 36 vs 36 + Ptx1-dsRNA, ****P* = 0.0002.

Fig. 7i, 7k Statistical analysis was performed using one-way ANOVA followed by *post hoc* Tukey's test:
1) Bassoon: C902 vs C902 + PITX2-siRNA, *P* = 0.9996; C902 vs C902-03, *****P* < 0.0001; C902-03 vs C902-03 + PITX2-siRNA, *****P* < 0.0001;
2) Homer1: C902 vs C902 + PITX2-siRNA, *P* = 0.1645; C902 vs C902-03, *****P* < 0.0001; C902-03 vs C902-03 + PITX2-siRNA, *****P* < 0.0001.

Fig. 8a Statistical analysis was performed using two-tailed unpaired Student's *t*-test:
1) *Fuzzy*$^{YY1\text{-}a}$: C902 vs C902-03, ***P* = 0.0033; C902 + BIND vs C902-03 + BIND, *P* = 0.4892; C902 + BIND-S vs C902-03 + BIND-S, **P* = 0.0487;
2) *Fuzzy*$^{YY1\text{-}b}$: C902 vs C902-03, ***P* = 0.0015; C902 + BIND vs C902-03 + BIND, *P* = 0.1240; C902 + BIND-S vs C902-03 + BIND-S, ***P* = 0.0053.

Fig. 8b-c Statistical analysis was performed using one-way ANOVA followed by *post hoc* Tukey's test:
1) Fuzzy: C902 vs C902-03, *****P* < 0.0001; C902-03 vs C902-03 + BIND, ****P* = 0.0006; C902-03 + BIND vs C902-03 + BIND-S, ***P* = 0.0029;
2) PITX2: C902 vs C902-03, ***P* = 0.0068; C902-03 vs C902-03 + BIND, **P* = 0.0177; C902-03 + BIND vs C902-03 + BIND-S, **P* = 0.0425.

**Fig. 8e, 8g** Statistical analysis was performed using one-way ANOVA followed by *post hoc* Tukey's test:<br>1) Bassoon: C902 vs C902 + BIND, *P* = 0.9978; C902 vs C902 + BIND-S, *P* = 0.9916; C902 vs C902-03, ****P < 0.0001; C902-03 vs C902-03 + BIND, ****P < 0.0001; C902-03 vs C902-03 + BIND-S, *P* = 0.9444;<br>2) Homer1: C902 vs C902 + BIND, *P* = 0.5986; C902 vs C902 + BIND-S, *P* = 0.5986; C902 vs C902-03, ****P < 0.0001; C902-03 vs C902-03 + BIND, ****P < 0.0001; C902-03 vs C902-03 + BIND-S, *P* = 0.7751.
**Supplementary Fig. 1b-e** Statistical analysis was performed using one-way ANOVA followed by *post hoc* Tukey's test:<br>1) Inturned: 180 vs C901-06, *P* = 0.9998; 180 vs C901-07, *P* > 0.9999; 180 vs C904-01, *P* = 0.9655; 180 vs C904-12, *P* = 0.9808; 840 vs C901-06, *P* = 0.8471; 840 vs C901-07, *P* = 0.9800; 840 vs C904-01, *P* > 0.9999; 840 vs C904-12, *P* > 0.9999; 841 vs C901-06, *P* = 0.8050; 841 vs C901-07, *P* = 0.5370; 841 vs C904-01, *P* = 0.1849; 841 vs C904-12, *P* = 0.2172;<br>2) Wdpcp: 180 vs C901-06, *P* = 0.9804; 180 vs C901-07, *P* > 0.9999; 180 vs C904-01, *P* = 0.9934; 180 vs C904-12, *P* > 0.9999; 840 vs C901-06, *P* > 0.9999; 840 vs C901-07, *P* = 0.9866; 840 vs C904-01, *P* > 0.9999; 840 vs C904-12, *P* = 0.9994; 841 vs C901-06, *P* = 0.9993; 841 vs C901-07, *P* = 0.9985; 841 vs C904-01, *P* > 0.9999; 841 vs C904-12, *P* > 0.9999;<br>3) Rsg1: 180 vs C901-06, *P* > 0.9999; 180 vs C901-07, *P* > 0.9999; 180 vs C904-01, *P* = 0.8502; 180 vs C904-12, *P* > 0.9999; 840 vs C901-06, *P* = 0.9988; 840 vs C901-07, *P* = 0.9981; 840 vs C904-01, *P* = 0.4692; 840 vs C904-12, *P* = 0.9974; 841 vs C901-06, *P* = 0.9913; 841 vs C901-07, *P* = 0.9880; 841 vs C904-01, *P* = 0.3603; 841 vs C904-12, *P* = 0.9856;<br>4) Jbts17: 180 vs C901-06, *P* = 0.9982; 180 vs C901-07, *P* > 0.9999; 180 vs C904-01, *P* > 0.9999; 180 vs C904-12, *P* > 0.9999; 840 vs C901-06, *P* = 0.8634; 840 vs C901-07, *P* = 0.9945; 840 vs C904-01, *P* = 0.9781; 840 vs C904-12, *P* = 0.9482; 841 vs C901-06, *P* = 0.2819; 841 vs C901-07, *P* = 0.6006; 841 vs C904-01, *P* = 0.4892; 841 vs C904-12, *P* = 0.4004.<br><br>**Supplementary Fig. 1f** Statistical analysis was performed using one-way ANOVA followed by *post hoc* Tukey's test: 180 vs I383T 0101, *P* > 0.9999; 180 vs I383T 0102, *P* > 0.9999; 180 vs M337V 0303, *P* = 0.9996; 180 vs M337V 0304, *P* > 0.9999; 180 vs M337V 0306, *P* = 0.8387; 840 vs I383T 0101, *P* > 0.9999; 840 vs I383T 0102, *P* > 0.9999; 840 vs M337V 0303, *P* > 0.9999; 840 vs M337V 0304, *P* = 0.9994; 840 vs M337V 0306, *P* = 0.9330; 841 vs I383T 0101, *P* = 0.9984; 841 vs I383T 0102, *P* = 0.9885; 841 vs M337V 0303, *P* = 0.9999; 841 vs M337V 0304, *P* = 0.9380; 841 vs M337V 0306, *P* = 0.9992.
**Supplementary Fig. 2b** Statistical analysis was performed using one-way ANOVA followed by *post hoc* Tukey's test: 3 vs 36, **P = 0.0064; 36 vs 92, **P = 0.0020; 3 vs 92, ****P < 0.0001.<br><br>**Supplementary Fig. 2c** Statistical analysis was performed using two-tailed unpaired Student's *t*-test: pUltra vs GA, *P* = 0.6029; pUltra vs GR, *P* = 0.6172; pUltra vs PR, *P* = 0.1962; Control-siRNA vs C9orf72-siRNA, *P* = 0.6508.<br><br>**Supplementary Fig. 2d** Statistical analysis was performed using two-tailed unpaired Student's *t*-test: Control-siRNA vs C9orf72-siRNA, *P = 0.0137.
**Supplementary Fig. 3e** Statistical analysis was performed using two-tailed unpaired Student's *t*-test: Control-siRNA vs YY1-siRNA, **P = 0.0018.

Supplementary Fig. 10c Statistical analysis was performed using one-way ANOVA followed by *post hoc* Tukey's test: 2 vs 2+YY1, $P = 0.8827$; 2 vs 66, $P = 0.9992$, 66 vs 66 + YY1, $P > 0.999$.

Supplementary Fig. 10d Statistical analysis was performed using two-tailed unpaired Student's *t*-test: elav vs elav + Pho, *$P = 0.0152$.

Supplementary Fig. 11a Statistical analysis was performed using one-way ANOVA followed by *post hoc* Tukey's test: 180 vs C901-06, **$P = 0.0031$; 180 vs C901-07, **$P = 0.0042$; 180 vs C904-01, ***$P = 0.0002$; 180 vs C904-12, ***$P = 0.0001$; 840 vs C901-06, **$P = 0.0038$; 840 vs C901-07, **$P = 0.0053$; 840 vs C904-01, ***$P = 0.0003$; 840 vs C904-12, ***$P = 0.0001$; 841 vs C901-06, **$P = 0.0056$; 841 vs C901-07, **$P = 0.0077$; 841 vs C904-01, ***$P = 0.0004$; 841 vs C904-12, ***$P = 0.0002$. Statistical analysis was performed using one-way ANOVA followed by *post hoc* Dunnett's test: C902 vs C902-02, ***$P = 0.0005$; C902 vs C902-03, **$P = 0.0023$.

Supplementary Fig. 11b Statistical analysis was performed using one-way ANOVA followed by *post hoc* Tukey's test: 180 vs C901-06, *$P = 0.0179$; 180 vs C901-07, **$P = 0.0017$; 180 vs C904-01, **$P = 0.0015$; 180 vs C904-12, *$P = 0.0273$; 840 vs C901-06, *$P = 0.0174$; 840 vs C901-07, **$P = 0.0017$; 840 vs C904-01, **$P = 0.0014$; 840 vs C904-12, *$P = 0.0265$; 841 vs C901-06, *$P = 0.0152$; 841 vs C901-07, **$P = 0.0015$; 841 vs C904-01, **$P = 0.0013$; 841 vs C904-12, *$P = 0.0231$. Statistical analysis was performed using one-way ANOVA followed by *post hoc* Dunnett's test: C902 vs C902-02, **$P = 0.0022$; C902 vs C902-03, *$P = 0.0378$.

Supplementary Fig. 11c Statistical analysis was performed using two-tailed unpaired Student's *t*-test: Control-siRNA vs PITX2-siRNA, *$P = 0.0295$.

Supplementary Fig. 11d Statistical analysis was performed using two-tailed unpaired Student's *t*-test: Control-siRNA vs CCND1-siRNA, ***$P = 0.0009$.

Supplementary Fig. 11e Statistical analysis was performed using one-way ANOVA followed by *post hoc* Tukey's test: Empty vector vs Empty vector + CCND1-siRNA, $P = 0.7491$; 2 vs 2 + CCND1-siRNA, $P = 0.6564$; 2 vs 66, **$P = 0.0028$; 66 vs 66 + CCND1-siRNA, $P = 0.1981$.

Supplementary Fig. 11f Statistical analysis was performed using two-tailed unpaired Student's *t*-test: Control-siRNA vs FOSL1-siRNA, ***$P = 0.0006$.

Supplementary Fig. 11g Statistical analysis was performed using one-way ANOVA followed by *post hoc* Tukey's test: Empty vector vs Empty vector + FOSL1-siRNA, $P = 0.9992$; 2 vs 2 + FOSL1-siRNA, $P = 0.9953$; 2 vs 66, *$P = 0.0168$; 66 vs 66 + FOSL1-siRNA, $P = 0.6873$.

Supplementary Fig. 11h Statistical analysis was performed using one-way ANOVA followed by *post hoc* Tukey's test: Empty vector vs Empty vector + PITX2, $P = 0.9854$; 2 vs 2 + PITX2, $P = 0.5631$; 2 vs 66, ***$P = 0.0003$; 66 vs 66 + PITX2, ***$P = 0.0001$.

Supplementary Fig. 12a Statistical analysis was performed using two-tailed unpaired Student's *t*-test: elav vs elav + Ptx1-dsRNA, *$P = 0.0146$.

Supplementary Fig. 12b Statistical analysis was performed using two-tailed unpaired Student's *t*-test: elav vs elav + Ptx1, *$P = 0.0146$.

**Supplementary Fig. 12c** Statistical analysis was performed using one-way ANOVA followed by *post hoc* Tukey's test: 3 vs 3 + Ptx1, $P = 0.9727$; 3 vs 36, **$P = 0.0036$; 36 vs 36 + Ptx1, ****$P < 0.0001$.<br><br>**Supplementary Fig. 12d** Statistical analysis was performed using Log-rank (Mantel-Cox) test: 3 vs 3 + Ptx1, $P = 0.4786$; 3 vs 36, ****$P < 0.0001$; 36 vs 36 + Ptx1, ****$P < 0.0001$.
**Supplementary Fig. 13b, 13d** Statistical analysis was performed using one-way ANOVA followed by *post hoc* Tukey's test:<br>1) Bassoon: C902 vs C902 + PITX2, $P = 0.5071$; C902 vs C902-03, ****$P < 0.0001$; C902-03 vs C902-03 + PITX2, ***$P = 0.0001$;<br>2) Homer1: C902 vs C902 + PITX2, $P = 0.4035$; C902 vs C902-03, ****$P < 0.0001$; C902-03 vs C902-03 + PITX2, **$P = 0.0038$.
