# Supplementary material for: Mutant GGGGCC RNA prevents YY1 from binding to Fuzzy promoter which stimulates Wnt/β-catenin pathway in C9ALS/FTD
Source: Nat Commun. 2023 Dec 18;14:8420. doi: 10.1038/s41467-023-44215-w (PMC10728118; doi:10.1038/s41467-023-44215-w)
Supplement: Supplementary file 3 — Reporting Summary [file 41467_2023_44215_MOESM3_ESM.pdf]

nature portfolio

Corresponding author(s): Ho Yin Edwin Chan, Kevin Talbot

Last updated by author(s): 2023/11/30

# Reporting Summary

Nature Portfolio wishes to improve the reproducibility of the work that we publish. This form provides structure for consistency and transparency in reporting. For further information on Nature Portfolio policies, see our Editorial Policies and the Editorial Policy Checklist.

## Statistics

For all statistical analyses, confirm that the following items are present in the figure legend, table legend, main text, or Methods section.

| n/a | Confirmed | |
|---|---|---|
| ☐ | ☒ | The exact sample size (*n*) for each experimental group/condition, given as a discrete number and unit of measurement |
| ☐ | ☒ | A statement on whether measurements were taken from distinct samples or whether the same sample was measured repeatedly |
| ☐ | ☒ | The statistical test(s) used AND whether they are one- or two-sided<br>*Only common tests should be described solely by name; describe more complex techniques in the Methods section.* |
| ☒ | ☐ | A description of all covariates tested |
| ☐ | ☒ | A description of any assumptions or corrections, such as tests of normality and adjustment for multiple comparisons |
| ☐ | ☒ | A full description of the statistical parameters including central tendency (e.g. means) or other basic estimates (e.g. regression coefficient) AND variation (e.g. standard deviation) or associated estimates of uncertainty (e.g. confidence intervals) |
| ☐ | ☒ | For null hypothesis testing, the test statistic (e.g. *F*, *t*, *r*) with confidence intervals, effect sizes, degrees of freedom and *P* value noted<br>*Give P values as exact values whenever suitable.* |
| ☒ | ☐ | For Bayesian analysis, information on the choice of priors and Markov chain Monte Carlo settings |
| ☒ | ☐ | For hierarchical and complex designs, identification of the appropriate level for tests and full reporting of outcomes |
| ☒ | ☐ | Estimates of effect sizes (e.g. Cohen's *d*, Pearson's *r*), indicating how they were calculated |

*Our web collection on statistics for biologists contains articles on many of the points above.*

## Software and code

Policy information about availability of computer code

| | |
|---|---|
| Data collection | All gels and blots were visualized using the Bio-rad ChemiDoc Touch Imaging System;<br>Thermal cycling was performed on a Bio-Rad CFX96 Real-time PCR detection system;<br>Confocal images were acquired on an Olympus IX-81 FV1000 confocal microscope or on a Leica TCS SP8 high speed imaging system;<br>All absorbance, luminescence and fluorescence readings were recorded on a Spark® multimode microplate reader. |
| Data analysis | The intensities of the DNA, RNA and protein bands were quantified using the ImageJ software version 1.52k;<br>Confocal images were analyzed using Olympus Fluoview software Version 4.2a or the Leica Application Suite X software;<br>All absorbance, luminescence and fluorescence readings were determined by the SparkControl software version 2.1;<br>The differential expressed gene lists were generated using the Partek Flow Genomic Analysis Software;<br>GraphPad Prism version 9.2.0 was used to plot data and perform statistical analysis;<br>The illustrations were created using Adobe Illustrator version 27.0.1 and BioRender.com. Assembly of panels in finalized figures was done using Adobe Illustrator version 27.0.1. |

For manuscripts utilizing custom algorithms or software that are central to the research but not yet described in published literature, software must be made available to editors and reviewers. We strongly encourage code deposition in a community repository (e.g. GitHub). See the Nature Portfolio guidelines for submitting code & software for further information.

## Data

Policy information about availability of data

All manuscripts must include a data availability statement. This statement should provide the following information, where applicable:

- Accession codes, unique identifiers, or web links for publicly available datasets
- A description of any restrictions on data availability
- For clinical datasets or third party data, please ensure that the statement adheres to our policy

Transcription factor binding sites were predicted using Promo (http://alggen.lsi.upc.es/cgi-bin/promo_v3/promo/promoinit.cgi?dirDB=TF_8.3), JASPAR (http://jaspar.genereg.net/) and Animal TFDB 3.0 (http://bioinfo.life.hust.edu.cn/AnimalTFDB/#!/) databases. The Fuzzy promoter sequence was withdrawn from GenBank under the accession number NG_032843.1 [https://www.ncbi.nlm.nih.gov/nuccore/NG_032843.1/]. The YY1 zinc finger domain amino acid sequences were withdrawn from GenBank under the accession numbers NP_003394.1 (human) [https://www.ncbi.nlm.nih.gov/protein/NP_003394.1], XP_009426747.2 (chimpanzee) [https://www.ncbi.nlm.nih.gov/protein/XP_009426747.2], NP_001091550.1 (cattle) [https://www.ncbi.nlm.nih.gov/protein/NP_001091550.1], XP_020955358.1 (pig) [https://www.ncbi.nlm.nih.gov/protein/XP_020955358.1], NP_775412.1 (rat) [https://www.ncbi.nlm.nih.gov/protein/NP_775412.1], NP_033563.2 (mouse) [https://www.ncbi.nlm.nih.gov/protein/NP_033563.2], NP_001116880.1 (frog) [https://www.ncbi.nlm.nih.gov/protein/NP_001116880.1] and NP_524630.1 (fruit fly) [https://www.ncbi.nlm.nih.gov/protein/NP_524630.1]. The public transcriptomic datasets used in this study are available in the GEO under accession codes GSE143743 [https://www.ncbi.nlm.nih.gov/geo/query/acc.cgi?acc=GSE143743] and GSE168831 [https://www.ncbi.nlm.nih.gov/geo/query/acc.cgi?acc=GSE168831]. All data supporting the findings of this study are presented within the main manuscript and Supplementary Information. Source data are provided with this paper.

## Human research participants

Policy information about studies involving human research participants and Sex and Gender in Research.

| | |
|---|---|
| Reporting on sex and gender | We used three iPSC lines from healthy individuals (2 female and 1 male), one isogenic control iPSC line (female), six iPSC lines from three C9 patients (1 female and 2 male) and five iPSC lines from two TDP-43 patients (2 male). The number do not allow for a meaningful sex-disaggregated analysis. Therefore, no sex-disaggregated analysis was performed. |
| Population characteristics | N/A |
| Recruitment | N/A |
| Ethics oversight | All iPSC lines were derived from human skin biopsy fibroblasts, collected under ethical approval granted by the South Wales Research Ethics Committee (WA/12/0186) in the James Martin Stem Cell Facility, University of Oxford, under standardized protocols. |

Note that full information on the approval of the study protocol must also be provided in the manuscript.

# Field-specific reporting

Please select the one below that is the best fit for your research. If you are not sure, read the appropriate sections before making your selection.

☒ Life sciences ☐ Behavioural & social sciences ☐ Ecological, evolutionary & environmental sciences

For a reference copy of the document with all sections, see nature.com/documents/nr-reporting-summary-flat.pdf

# Life sciences study design

All studies must disclose on these points even when the disclosure is negative.

| | |
|---|---|
| Sample size | No sample size calculation was performed for this work. The number of iPSC lines/differentiation replicates used in this study represents common practice in the field (Ababneh et al., 2020, Hum Mol Genet; Dafinca et al., 2020, Stem Cell Rep). Unless specified otherwise n = 3 biologically independent experiments. The data met the assumptions for each statistical test performed. |
| Data exclusions | No data exclusions was done. |
| Replication | Experimental data were collected from at least three independent trials. Number of biological replicates of cell- or animal-based experiments were described in the figure legends of the manuscript. |
| Randomization | The cells and animals were randomly assigned for experiments. |
| Blinding | Investigators who conduct the experiments were blinded to group allocation as well as the allocation sequence. |

# Reporting for specific materials, systems and methods

We require information from authors about some types of materials, experimental systems and methods used in many studies. Here, indicate whether each material, system or method listed is relevant to your study. If you are not sure if a list item applies to your research, read the appropriate section before selecting a response.

## Materials & experimental systems

| n/a | Involved in the study |
|---|---|
| ☐ | ☒ Antibodies |
| ☐ | ☒ Eukaryotic cell lines |
| ☒ | ☐ Palaeontology and archaeology |
| ☐ | ☒ Animals and other organisms |
| ☒ | ☐ Clinical data |
| ☒ | ☐ Dual use research of concern |

## Methods

| n/a | Involved in the study |
|---|---|
| ☒ | ☐ ChIP-seq |
| ☒ | ☐ Flow cytometry |
| ☒ | ☐ MRI-based neuroimaging |

## Antibodies

Antibodies used

The primary antibodies used for immunocytochemistry were anti-PITX2 (1:200, H00005308-M01, Novus Biologicals, Centennial, CO, USA), anti-Tau (1:500, ab75714, Abcam, Cambridge, MA, USA), anti-Bassoon (1:200, ab110426, Abcam), anti-MAP2 (1: 1,000, ab5392, Abcam), anti-Homer1 (1:200, ab97593, Abcam), anti-YY1 (1:200, ab109237, Abcam), anti-YY1 (1:200, 22156-1-AP, Proteintech), anti-hnRNP H (1:200, ab10374, Abcam), anti-GFP (1:500, 632381, Takara Bio Inc., Shiga, Japan), and anti-Tuj1 (1:1,000, 801202, BioLegend, San Diego, CA, USA). Secondary antibodies used were Alexa Fluor 488 Goat anti-Chicken IgY H&L (1:500, ab150169, Abcam), Alexa Fluor 594 Donkey anti-Mouse IgG (H+L) (1:500, A-21203, Thermo Fisher Scientific), Alexa Fluor 488 Donkey anti-Mouse IgG (H+L) (1:500, A-21202, Thermo Fisher Scientific), and Alexa Fluor 647 Donkey anti-Rabbit IgG (H+L) (1:500, A-31573, Thermo Fisher Scientific).

The primary antibodies used for immunoblotting were anti-Fuzzy (1:1,000, ab111842), anti-YY1 (1:1,000, ab109237), anti-CCND1 (1:1,000, ab134175), anti-FOSL1 (1:1,000, ab124722), anti-PITX2 (1:1,000, ab32832), anti-β-tubulin (1:2,000, ab6046) from Abcam, anti-C9orf72 (1:1,000, GTX632041) from Genetex (Irvine, CA, USA), anti-YY1 (1:1,000, MAB3784) from R&D Systems, Inc. and anti-mCherry (1:1,000, NBP1-96752) from Novus Biologicals. Secondary antibodies used were HRP-conjugated goat anti-rabbit IgG (H + L) (11-035-045, 1:5,000) and HRP-conjugated goat anti-mouse IgG (H + L) (115-035-062, 1:10,000) from Jackson ImmunoResearch (West Grove, PA, USA).

Four micrograms of anti-YY1 (22156-1-AP, Proteintech) or anti-mCherry (ab167453, Abcam) antibody was used for the ChIP.

Validation

The anti-PITX2 antibody (H00005308-M01, Novus Biologicals) detects human PITX2 protein and is suitable for ICC (https://www.novusbio.com/products/pitx2-antibody-2g6_h00005308-m01);
The anti-Tau antibody (ab75714, Abcam) detects human Tau protein and is suitable for ICC (https://www.abcam.com/products/primary-antibodies/tau-antibody-ab75714.html);
The anti-Bassoon antibody (ab110426, Abcam) detects human Bassoon protein (https://www.abcam.com/products/primary-antibodies/bassoonbsn-antibody-ab110426.html), and we have demonstrated in our paper that it is suitable for ICC;
The anti-MAP2 antibody (ab5392, Abcam) detects human MAP2 protein and is suitable for ICC (https://www.abcam.com/products/primary-antibodies/map2-antibody-ab5392.html);
The anti-Homer1 antibody (ab97593, Abcam) detects human Homer1 protein (https://www.abcam.com/products/primary-antibodies/homer1-antibody-ab97593.html), and we have demonstrated in our paper that it is suitable for ICC;
The anti-YY1 antibody (ab109237, Abcam) detects human YY1 protein and is suitable for ICC and WB (https://www.abcam.com/products/primary-antibodies/yy1-antibody-epr4652-nuclear-loading-control-ab109237.html);
The anti-YY1 antibody (22156-1-AP, Proteintech) detects human YY1 protein and is suitable for ICC and ChIP (https://www.ptglab.com/products/YY1-Antibody-22156-1-AP.htm);
The anti-hnRNP H antibody (ab10374, Abcam) detects human hnRNP H protein and is suitable for ICC (https://www.abcam.com/products/primary-antibodies/hnrnp-h-antibody-ab10374.html);
The anti-GFP antibody (632381, Takara) detects GFP protein (https://www.takarabio.com/products/antibodies-and-elisa/fluorescent-protein-antibodies/green-fluorescent-protein-antibodies), and we have demonstrated in our paper that it is suitable for ICC;
The anti-Tuj1 antibody (801202, BioLegend) detects human Tuj1 protein and is suitable for ICC (https://www.biolegend.com/en-us/sean-tuckers-tests/purified-anti-tubulin-beta-3-tubb3-antibody-11580?GroupID=GROUP686);
The anti-Fuzzy antibody (ab111842, Abcam) detects human Fuzzy protein and is suitable for WB (https://www.abcam.com/products/primary-antibodies/fuz-antibody-ab111842.html);
The anti-CCND1 antibody (ab134175, Abcam) detects human CCND1 protein and is suitable for WB (https://www.abcam.com/products/primary-antibodies/cyclin-d1-antibody-epr2241-c-terminal-ab134175.html);
The anti-FOSL1 antibody (ab124722, Abcam) has been discontinued, but we have demonstrated in our paper that it detects human FOSL1 protein at correct size;
The anti-PITX2 antibody (ab32832, Abcam) has been discontinued, but we have demonstrated in our paper that it detects human PITX2 protein at correct size;
The anti-β-tubulin antibody (ab6046, Abcam) detects human β-tubulin protein and is suitable for WB (https://www.abcam.com/products/primary-antibodies/beta-tubulin-antibody-loading-control-ab6046.html);
The anti-C9orf72 antibody (GTX632041, Genetex) detects human C9orf72 protein and is suitable for WB (https://www.genetex.com/Product/Detail/C9orf72-antibody-GT779/GTX632041);
The anti-YY1 antibody (MAB3784, R&D Systems, Inc.) detects human YY1 protein and is suitable for WB (https://www.bio-

techne.com/p/antibodies/human-mouse-yy1-antibody-854847_mab3784);
The anti-mCherry antibody (NBP1-96752, Novus Biologicals) detects mCherry protein and is suitable for WB (https://www.novusbio.com/products/mcherry-antibody-1c51_nbp1-96752);
The anti-mCherry antibody (ab167453, Abcam) detects mCherry protein (https://www.abcam.com/products/primary-antibodies/mcherry-antibody-ab167453.html), and we have demonstrated in our paper that it is suitable for ChIP.

## Eukaryotic cell lines

Policy information about cell lines and Sex and Gender in Research

| | |
|---|---|
| Cell line source(s) | SK-N-MC cells, ATCC®, HTB-10TM;<br>All iPSC lines were derived from human skin biopsy fibroblasts, collected under ethical approval granted by the South Wales Research Ethics Committee (WA/12/0186) in the James Martin Stem Cell Facility, University of Oxford, under standardized protocols.<br>Healthy control iPSC lines: Control180, Control840 and Control841;<br>Isogenic control iPSC line: Isogenic controlC902;<br>C9 patient iPSC lines: C9ALS/FTD patientC901-06, C9ALS/FTD patientC901-07, C9ALS/FTD patientC902-02, C9ALS/FTD patientC902-03, C9ALS patientC904-01, C9ALS patientC904-12;<br>TDP-43 patient iPSC lines: ALS patient TARDBPI383T 0101, ALS patient TARDBPI383T 0102, ALS patient TARDBPM337V 0303, ALS patient TARDBPM337V 0304, ALS patient TARDBPM337V 0306 |
| Authentication | The SK-N-MC cell line is available at ATCC. All iPSCs were generated in-house and have been published and characterised extensively before (Ababneh et al., 2020, Hum Mol Genet; Dafinca et al., 2020, Stem Cell Rep). No additional authentication of these cell lines was performed in our laboratory. |
| Mycoplasma contamination | Cells are negative for mycoplasma contamination. |
| Commonly misidentified lines<br>(See ICLAC register) | No commonly mis-identified cell lines were used, according to the ICLAC Register. |

## Animals and other research organisms

Policy information about studies involving animals; ARRIVE guidelines recommended for reporting animal research, and Sex and Gender in Research

| | |
|---|---|
| Laboratory animals | All Drosophila melanogaster stocks and genetic crosses were maintained on the cornmeal medium in a 25°C incubator. The elavGS-Gal4 (43642), UAS-(GGGGCC)3 (58687), and UAS-(GGGGCC)36 (58688) lines were obtained from Bloomington Drosophila Stock Center (Bloomington, IN, USA). The UAS-Ptx1-dsRNAKK108574 (107785) line was obtained from Vienna Drosophila RNAi Center (Vienna, Austria). The UAS-Pho (F000151) and UAS-Ptx1 (F003469) lines were obtained from FlyORF (Zurich, Switzerland). The adult Drosophila melanogaster were used in the climbing assays: Fig. 6d (14 days post eclosion), Fig. 7f (14 days post eclosion), Supplementary Fig. 12c (10 days post eclosion). For the survival assay, the survival rate of flies was recorded daily until flies from each experimental group were all dead. |
| Wild animals | No wild animals were involved in this study. |
| Reporting on sex | Both male and female flies were used in this study. |
| Field-collected samples | No field-collected samples were involved in this study. |
| Ethics oversight | All animal procedures were approved by the CUHK Animal Experimentation Ethics Committee (and their care was in accordance with the institutional guidelines). |

Note that full information on the approval of the study protocol must also be provided in the manuscript.
